# Lipid Regulation of Mechanosensitive Ion Channels

**DOI:** 10.3390/ijms27041984

**Published:** 2026-02-19

**Authors:** Yurou Cai, Claudia Bauer, Jian Shi

**Affiliations:** Leeds Institute of Cardiovascular and Metabolic Medicine, School of Medicine, University of Leeds, Leeds LS2 9JT, UK; umyc@leeds.ac.uk (Y.C.); c.bauer@leeds.ac.uk (C.B.)

**Keywords:** mechanosensitive ion channels, regulation, lipids, fatty acids, piezo channel, Msc channel, TRP channel, K2P channel

## Abstract

Mechanosensitive ion channels (MSCs) are fundamental transducers that convert mechanical forces into electrochemical signals, enabling cells to regulate processes such as Ca^2+^ homeostasis, migration, proliferation, and adhesion. Located in both plasma and organellar membranes, MSCs, including Piezos, TRPs, K2Ps, MscL, and MscS families exhibit diverse ion selectivity, gating mechanisms and physiological roles. Emerging evidence demonstrates that lipids are dynamic regulators of MSC activation, sensitivity, and kinetics. Endogenous membrane lipids such as cholesterol, phospholipids, sphingolipids and fatty acids modulate MSC behavior by altering bilayer tension, curvature, stiffness and protein–lipid interactions. Exogenous lipids, including dietary fatty acids and lipid-derived metabolites, influence MSCs by modifying membrane physical properties or engaging specific lipid-binding sites on channel proteins. These interactions shape fundamental biological processes and contribute to disease mechanisms in cardiovascular dysfunction, neurological disorders, metabolic disease, and cancer. Despite significant progress, the molecular principles by which lipids regulate MSC conformational transitions and force sensing remain incompletely defined. This review synthesizes current knowledge on endogenous and exogenous lipid modulation of MSCs, integrating structural, computational and electrophysiological insights to highlight emerging therapeutic opportunities targeting lipid–mechanotransduction interfaces.

## 1. Introduction

**Figure 1:**
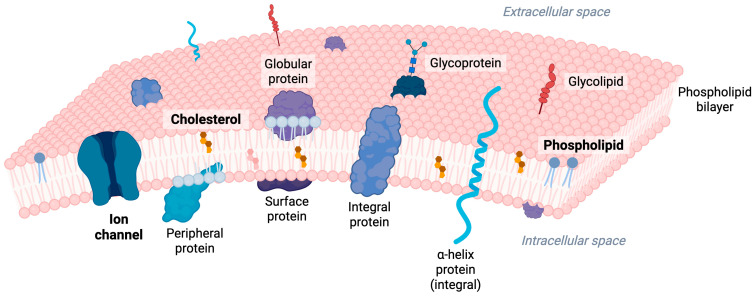
Schematic representation of the eukaryotic plasma membrane and its molecular components. Illustration of the plasma membrane structure highlighting the phospholipid bilayer and associated macromolecules. Membrane components include phospholipids and cholesterol, which regulate bilayer fluidity and stability; integral proteins such as transmembrane α-helix proteins, ion channels, and glycoproteins; peripheral and surface proteins located on the cytoplasmic or extracellular leaflet; and glycolipids embedded in the outer leaflet. Together, these elements contribute to membrane integrity, selective permeability, cell signaling, and interactions with the extracellular environment. Created in BioRender. Cai, Y. (2026) https://BioRender.com/4lyq9j2.

Mechanosensitive ion channels (MSCs), located in the plasma membrane and within the membranes of various intracellular organelles [1] (Figure 1), constitute an essential family of pore-forming proteins that detect and transduce mechanical stimuli into electrochemical signals, thereby enabling cells to sense and respond to mechanical forces [1,2]. Mechanical cues can trigger the activation of MSCs, permitting ions to pass through the membrane in which each channel resides [3]. In eukaryotic cells, many MSCs exhibit a range of ion selectivity, from non-selective conductance to cation-preferring permeability, facilitating the passage of Ca^2+^, K^+^, and Na^+^ [4,5]. At the cell surface, Piezo channels localize to the plasma membrane and transduce mechanical stretch into Ca^2+^ entry [6]. MSCs also operate on intracellular organelles: channels on the endoplasmic reticulum (e.g., Polycystin-2(PC2) [7] and lysosomes (e.g., transmembrane protein 63 (TMEM63) [8], can release ions from the organelle lumen into the cytosol or, depending on electrochemical gradients, permit ion entry into the organelle. In contrast, bacterial channels such as mechanosensitive channel small conductance (MscS) and mechanosensitive channel large conductance (MscL) are non-selective pores that permit both cations and anions to pass under osmotic stress [3]. This diversity in ion selectivity highlights that MSCs range from highly cation-selective to broadly non-selective, depending on their structure and physiological role.

By coupling mechanical stimulation to Ca^2+^-dependent signaling pathways, MSCs play a critical role in Ca^2+^ homeostasis, which in turn governs a broad array of downstream cellular behaviors [9]. Beyond regulating intracellular Ca^2+^ dynamics, MSCs contribute to key processes such as cell proliferation, migration, and adhesion [10,11,12]. Extensive research has demonstrated that MSCs are involved in diverse physiological processes, including touch sensation, hearing, vascular tone regulation, and osmoregulation, whereas their dysfunction has been associated with pathological conditions such as chronic pain, cardiovascular diseases, and cancer metastasis [13,14,15].

Given their pivotal roles in both health and disease, understanding the mechanisms that regulate MSC activity is essential. As integral membrane proteins, MSCs are embedded within the lipid bilayer, where surrounding lipids serve not merely as a passive structural framework but as active modulators of channel function [16,17] (Figure 1). Increasing evidence indicates that the lipid environment of MSCs is a key determinant of their gating properties, sensitivity, and kinetics [17,18].

Membrane lipid composition is highly dynamic, varying between all organisms, including tissues, cells and organelles, also through the life cycle [19,20]. It can also be remodeled in response to environmental changes, such temperature, pressure and pH, to maintain optimal membrane properties [19,21,22,23]. Under the mechanical cues, the chemical composition of the bilayer would be altered, affecting its mechanical properties, such as its thickness or curvature [24,25]. Changes to its physical properties can influence the behavior of proteins associated with the membrane, thereby fine-tuning MSC function.

Beyond endogenous membrane lipid composition, several exogenous lipids have been shown to modulate MSC activity. Fatty acids and lipid-derived bioactive molecules can alter membrane properties and consequently influence the gating of channels, including Piezo1 [18], Piezo2 [26] and TRPV4 channels [27], indicating that lipid–MSC regulation is governed not only by membrane lipid composition but also by exogenous lipids derived from nutritional and environmental sources.

Although progress has been made in demonstrating lipid influences on MSC activity, the molecular mechanisms remain incompletely understood. Importantly, the therapeutic implications of lipid–MSC interactions are only beginning to be explored. This review summarizes current knowledge on how lipids, including both endogenous and exogenous ones, regulate diverse MSCs. We integrate evidence from structural biology, electrophysiology, and computational modelling to provide molecular-level insights and explore the therapeutic potential of mechanosensing modifications by lipids.

## 2. Mechanosensitive Ion Channels: An Overview

MSCs are widely distributed across phylogeny, from bacteria to humans [28], highlighting their evolutionary significance and fundamental importance for cellular homeostasis. The first mechanosensitive ion channel identified in eukaryotes was described by Guharay and Sachs [29], who provided patch-clamp evidence of stretch-activated channels in embryonic chick skeletal muscle. Subsequently, Martinac, Buechner [30] recorded mechanosensitive channel activity in giant spheroplasts derived from *Escherichia coli*, demonstrating that bacteria also possess stretch-activated ion channels. These early discoveries established that mechanosensitive channels are conserved across diverse life forms and laid the groundwork for subsequent studies into their molecular identity, structural basis, and physiological functions.

Several criteria have been proposed to confirm whether a candidate protein can be considered as an MSC [2,31]. The channel must be expressed in mechanosensory cells and appropriately localized to the membrane domain where mechanical forces are detected. Its presence should be essential for the mechanically evoked response, without being required for normal mechanoreceptor development or unrelated downstream signaling. Loss-of-function should, therefore, diminish or abolish the mechanically evoked response; whereas reintroduction or ectopic expression, ideally in a heterologous system or reconstituted lipid bilayer, should restore or confer mechanically gated activity [4]. Additional evidence for a direct role can be obtained by altering the channel protein itself, for example, mutations that change ion selectivity, conductance, or gating kinetics and demonstrating corresponding changes in the mechanically evoked response; furthermore, the gating behavior observed in recombinant systems should recapitulate that in the native context, confirming the channel as an integral component of the mechanotransduction machinery [32].

Although only a limited number of MSCs have been discovered that meet all the established criteria, their discovery remains a critical first step towards understanding mechanotransduction processes in vivo. Identification and characterization of temperature-activated transient receptor potential (TRP) channels have improved understanding of thermal sensing mechanisms in both vertebrates and invertebrates [33], while the discovery of the Piezo family has provided a molecular basis for how cells detect and transduce mechanical stimuli into biochemical signals [6].

**Figure 2:**
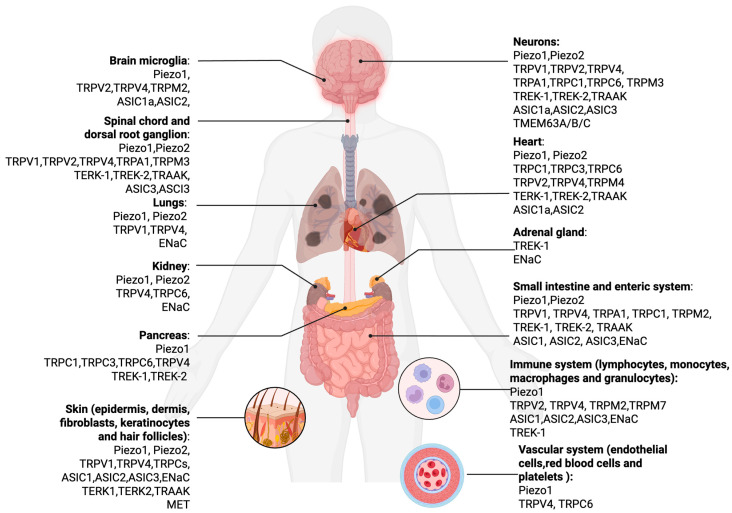
Expression of mechanosensitive ion channels across human tissues. Schematic representation of the distribution of mechanosensitive ion channels in the human body. Distinct tissues and cell types express a range of mechanosensitive channels, including Piezo1/2, members of the TRP (TRPV1–4, TRPA1, TRPC1/3/6, TRPM2/3/4/7), K2P (TREK-1, TREK-2, TRAAK), and ASIC (ASIC1a, ASIC1c, ASIC2, ASIC3) families, as well as ENaC, TMEM63A/B/C, and MET [34,35,36,37]. Expression patterns are indicated for brain microglia, neurons, spinal cord and dorsal root ganglion, lungs, heart, kidney, pancreas, adrenal gland, small intestine and enteric system, immune cells, vascular system and skin. Created in BioRender. Cai, Y. (2026) https://BioRender.com/4lyq9j2.

Recent advances have revealed remarkable diversity among MSCs in both structure and function. These channels can be broadly classified based on their molecular families. Structural studies have identified distinct channel families such as the Piezos, TRP, osmolality-induced [Ca^2+^] increase (OSCA)/TMEM63, Two-pore domain potassium (K2P), degenerin (DEG)/epithelial sodium channel (ENaC), and MscL/MscS families, each with unique architectures and ion selectivity. Their widespread expression across human tissues is illustrated in Figure 2 (excluding MscL/MscS families), which highlights the presence of these channels in organs such as the brain, heart, lungs, kidney, and skin, underscoring their essential roles in mechanotransduction and physiological homeostasis.

## 3. Activation Mechanisms of Mechanosensitive Ion Channels

**Figure 3:**
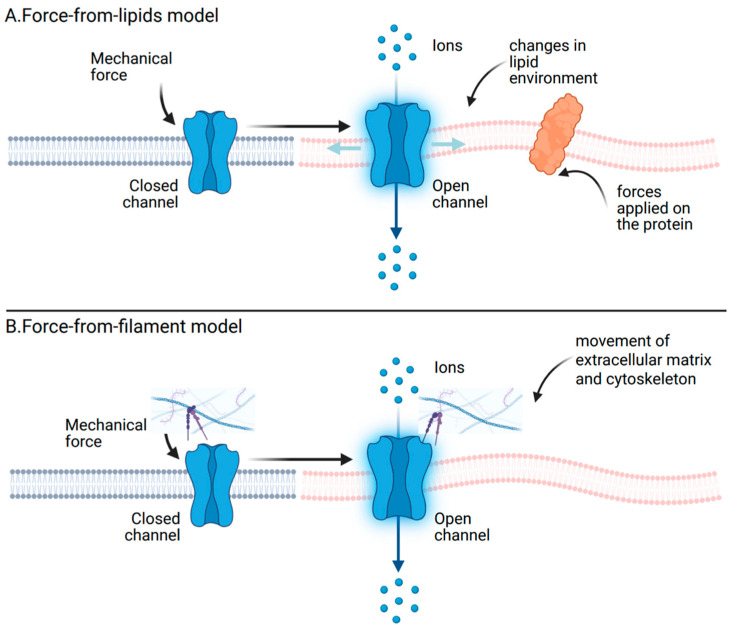
Models of mechanosensitive ion channel gating. Two primary mechanisms by which mechanical forces activate mechanosensitive ion channels are illustrated. (**A**) Force-from-lipids model: mechanical perturbations within the lipid bilayer alter membrane tension, leading to conformational changes in the channel protein and subsequent pore opening, enabling ion flux. (**B**) Force-from-filament model: mechanical forces are transmitted through cytoskeletal or extracellular matrix connections to the channel, inducing channel opening and ion conduction. Together, these models describe the fundamental pathways through which cells convert mechanical stimuli into ionic signals. Created in BioRender. Cai, Y. (2026) https://BioRender.com/4lyq9j2.

Mechanosensitive ion channels can be activated by two principal gating mechanisms. In the force-from-lipid (FFL) model, mechanical tension within the lipid bilayer is directly transmitted to the channel, inducing conformational changes that open the pore [38,39]. In contrast, the force-from-filament (FFF) model proposes that gating is mediated by mechanical tethers linking the channels to cytoskeletal or extracellular components, which exert force on the channel and modulate the conformation of its gate [40]. The key distinction between these two paradigms lies in the source of force transmission: force-from-lipid relies solely on bilayer mechanics, whereas force-from-filament requires additional molecular components to couple external forces to channel gating [41] (Figure 3).

The FFL principle originated from patch-clamp studies of the bacterial mechanosensitive channels MscS and MscL [41]. Remarkably, purified MscL obtained by cell-free expression and reconstituted into liposome patches retained intrinsic mechanosensitive activity. As the first mechanosensitive channel to be cloned and expressed in vitro, it evidenced that gating can be driven solely by lipid bilayer tension, independent of accessory proteins or cytoskeletal elements [42,43].

However, this property is not unique to MscL. Several eukaryotic mechanosensitive channels, including Piezo1 [44,45], K2P [46] and OSCA channels, have likewise been shown to retain mechanosensitivity in purified reconstituted systems, even in the absence of cytoskeletal or extracellular components. Brohawn, Su [46] purified human TRAAK and zebrafish TREK1 channels expressed in Pichia pastoris, reconstituted them into phosphatidylcholine bilayers, and demonstrated mechanosensitive currents in inside-out patch recordings from proteoliposomes, with current amplitudes scaling with the protein-to-lipid ratio. Cox, Bae [44] reported that Piezo1 could be activated in membrane blebs with lower pressure thresholds than in intact cells, and that this activation was unaffected by disrupting actin polymerization (cytochalasin D) or microtubule polymerization (colchicine), supporting the absence of cytoskeletal tethering in bleb membranes. Syeda, Florendo [45] further reconstituted purified Piezo1 into droplet lipid bilayers of symmetric composition and recorded channel activity. Mechanical activation was achieved by generating an osmotic gradient via mannitol injection into the cis monolayer, which induced an imbalance in osmotic pressure and altered the trans bilayer tension profile. In the absence of such osmotic strain, no activity was detected. Moreover, this response was specific to Piezo1 and mechanosensitive channels, as the non-mechanosensitive K^+^ channel KcsA showed no activation under identical conditions.

FFF principle requires the involvement of extracellular matrix and cytoskeleton [47], and has been well characterized in several mechanosensitive channel systems, such as mechanoelectrical transduction (MET) [48], TRP [49,50,51] and Piezo families [44]. In MET channels of hair cells, gating is mediated by the tip link, an extracellular filament that transmits mechanical force directly to the channel complex. When deflection of the stereocilia applies tension to the tip link, the MET channels open, permitting a rapid influx of K^+^ and Ca^2+^ into the hair bundle and thereby altering the receptor potential [48]. Similarly, certain TRP channels, such as TRPV4 [50] and TRPA1 [51], can be indirectly activated by external forces. Mechanical forces transmitted through β1 integrins trigger an exceptionally rapid Ca^2+^ influx via TRPV4 channels [50]. Notably, the activation of TRPV4 is attributable to mechanical distortion of the focal adhesion cytoskeletal framework, rather than to deformation of the plasma membrane lipid bilayer or the underlying cortical cytoskeleton [50]. Gaub and Müller [52] developed a method using atomic force microscopy (AFM) to mechanically stimulate Piezo1 channels in live animal cells, simultaneously monitoring receptor activation through real-time functional Ca^2+^ imaging. Their findings confirmed that in the absence of extracellular matrix (ECM) proteins, the Piezo1 channels had confined sensitivity to mechanical forces pushing at the cell membrane. However, when combined with Matrigel, a composite of ECM proteins, channels demonstrated increased response.

**Figure 4:**
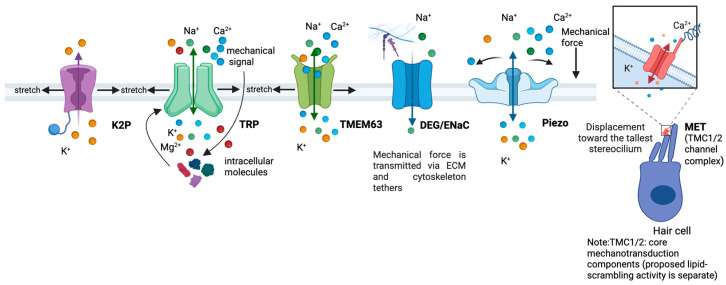
Representative families of mechanosensitive ion channels and their modes of activation. Schematic illustration of major mechanosensitive ion channel families, including K2P, TRP, TMEM63/OSCA, DEG/ENaC, Piezo, and hair-cell MET channels and the mechanisms through which they transduce mechanical stimuli into ionic signals. In cochlear hair cells, TMC1/2 function as essential components of the MET channel complex. TMC family proteins may also exhibit lipid-scrambling activity; this function is mechanistically distinct from their established role in mechanosensory transduction. Created in BioRender. Cai, Y. (2026) https://BioRender.com/4lyq9j2.

The two models, FFL and FFF, are not necessarily exclusive alternatives. Rather than representing a strict dichotomy, mechanosensory transduction is better understood as emerging from the coordinated and dynamic interplay among lipid bilayers, membrane-associated scaffold proteins, the cytoskeleton and extracellular matrix [16,47,52]. Figure 4 illustrates the major classes of mechanosensitive ion channels and the activation mechanisms through which mechanical forces are converted into ionic signals.

## 4. Regulation of MSCs by Different Lipids

MSCs comprise multiple structurally and functionally distinct families that enable cells to sense and respond to mechanical stimuli. Table 1 summarizes the major MSC families, representative members, and their defining functional characteristics, spanning organisms from bacteria to mammals and underpinning processes such as osmotic regulation, mechanosensation, hearing, touch, vascular physiology, and acid sensing.

### 4.1. Piezo Family

#### 4.1.1. Overview of the Piezo Family

The Piezo family comprises mechanosensitive ion channels in which mechanical stimuli act as gating signals, inducing the opening of a non-selective cation-permeable pore that allows the passage of ions such as Ca^2+^, K^+^, Na^+^ and Mg^2+^ [34,53,54,55,56].

Two isoforms, Piezo1 and Piezo2, have been identified in vertebrates [6]. Piezo1 is widely expressed in non-sensory tissues [57] including the lungs, bladder, and skin, as well as in the cardiovascular and nervous systems [57,58,59], where it plays essential roles in diverse physiological processes. In the vascular system, Piezo1 functions as a sensor of hemodynamic forces, such as shear stress and blood flow, thereby contributing to vascular tone regulation, blood pressure control, and the maintenance of cardiac structural and functional homeostasis [60,61,62]. Notably, global deletion of Piezo1 in mice results in mid-gestational embryonic lethality due to severe defects in vascular development and impaired endothelial responses to shear stress, demonstrating that Piezo1-mediated mechanotransduction is essential for embryonic vascular maturation and survival [63]. In the urinary bladder, Piezo1 functions as a mechanosensor that detects bladder wall stretch, initiating Ca^2+^ influx and promoting substantial ATP release [64]. Dual Piezo1/2 knockout mice exhibit the most severe phenotype, characterized by markedly reduced urothelial responses to mechanical stimulation, diminished ATP release, and bladder hypoactivity in anesthetized females [65]. Within the central nervous system (CNS), Piezo1 is expressed in neurons, astrocytes, oligodendrocytes, microglia, and vascular components. Neuronal Piezo1 channels contribute to developmental processes such as axonal growth and synaptogenesis [66,67]. In astrocytes, Piezo1 activation can trigger intracellular calcium waves, promoting gliotransmitter release [68,69], while in oligodendrocytes, Piezo1-mediated mechanotransduction participates in regulating myelination [70].

In contrast, Piezo2 is predominantly expressed in sensory neuron systems [71]. It is highly enriched in peripheral sensory neurons, including dorsal root ganglion (DRG) and trigeminal ganglion neurons, while it mediates gentle touch, vibration sensing, and facial mechanosensation [6,72]. In the skin, Piezo2 is present in Merkel cells, enabling fine touch discrimination [73,74]. It is also expressed in proprioceptive neurons innervating muscle spindles and Golgi tendon organs, providing feedback on muscle stretch and tension for body position awareness [75]. Viscerally, Piezo2 is found in vagal sensory neurons of the nodose and jugular ganglia, detecting airway stretch for respiratory control [76], as well as in bladder and gastrointestinal sensory neurons, where it senses organ distension [77]. Piezo1 and Piezo2 constitute a complementary mechanosensory system that is fundamental to homeostatic regulation and sensory perception in humans.

Structurally, Piezo proteins are exceptional among ion channels for their large size and the absence of sequence homology with other known ion channel families. Their architecture was identified through cryo-electron microscopy (cryo-EM), revealing a distinctive three-bladed, propeller-like configuration. Each subunit contributes to the formation of a central ion-conducting pore capped by an extracellular dome-like structure [78,79,80]. Much of the foundational understanding derives from near-atomic-resolution structures of mouse Piezo1, which provided the first detailed view of the architectural features underlying mechanogating [80].

The mouse Piezo1 (mPiezo1) protein comprises 2547 amino acid residues, whereas mouse Piezo2 (mPiezo2) contains 2822 residues, sharing approximately 42% sequence identity with mPiezo1 [81]. Earlier functional and predictive studies suggested that Piezo2 shares a similar trimeric architecture with Piezo1 [82]. This has since been confirmed by high-resolution cryo-electron microscopy, which revealed that Piezo2 assembles into a conserved trimeric, propeller-like structure characteristic of Piezo channels, comprising a total of 114 transmembrane helices (38 per protomer) [81]. Despite the divergence between mouse Piezo1 and Piezo2, the human and mouse orthologues of both channels are highly conserved, exhibiting more than 90% sequence identity. This high level of conservation indicates that structural insights derived from mouse Piezo1 are broadly applicable to human Piezo channels [72].

Recent near-atomic-resolution cryo-EM structures of human Piezo1 (hPiezo1), including both the fast-inactivating wild-type channel and slow-inactivating channelopathy mutants, have provided important insights into the structural basis of Piezo1 gating and inactivation [83]. These findings reveal that hPiezo1 adopts a more flattened and extended conformation than the more curved architecture observed in mouse Piezo1. The auxiliary, multi-lapidated subunit MDFIC binds laterally to the pore module of hPiezo1, similar to its interaction with mouse Piezo1, and shifts the channel into a more curved and extended state [83].

Activation of Piezo channels results in a rapid cation influx, occurring within milliseconds [55,84], with permeability to Ca^2+^ as well as monovalent ions such as Na^+^, which subsequently triggers downstream signaling pathways that regulate a wide range of physiological processes. Furthermore, the characteristic rapid activation and inactivation kinetics of the Piezo family are largely conserved across diverse cell types, with only minor variations observed [85,86]. Although Piezo channels are mainly activated by mechanical stimuli, several pharmacological molecules have been identified to modulate their activity.

Small-molecule modulators have been valuable tools for probing Piezo1 function. The first synthetic agonist, Yoda1, was identified through high-throughput screening and selectively activates Piezo1 over Piezo2 [87]. Although widely used, Yoda1 has limitations, including poor solubility and modest potency. Subsequent efforts yielded Yoda2, a Yoda1 analogue with improved efficacy toward human Piezo1 [88]. Further Piezo1-selective activators, including Jedi1 and Jedi2, were identified through high-throughput screening. These compounds lack structural similarity to Yoda1 and appear to engage distinct channel regions, highlighting multiple ligand-sensitive sites within Piezo1 [89]. In contrast to these activators, the spider venom peptide GsMTx4 functions as an antagonist of both Piezo1 and Piezo2 [90]. Its action is thought to arise from interactions with the lipid bilayer that reduce effective membrane tension transmitted to the channel [91].

Loss-of-function and gain-of–function mutations in Piezo channels have been associated with different human diseases, including hereditary xerocytosis, generalized lymphatic dysplasia, and distal arthrogryposis type 3 and type 5 [92]. These genetic associations underscore the essential roles of Piezo channels in mechanotransduction and highlight their potential as therapeutic targets.

#### 4.1.2. Lipid-Mediated Modulation of Piezo Channels

Piezo channel gating is regulated by the force-to-lipid mechanism, whereby forces within the lipid bilayer induce conformational changes that open the channel [93]. This suggests that Piezo1 activity likely depends on both global impacts on membrane physical characteristics and specific lipid interactions.

**Figure 5:**
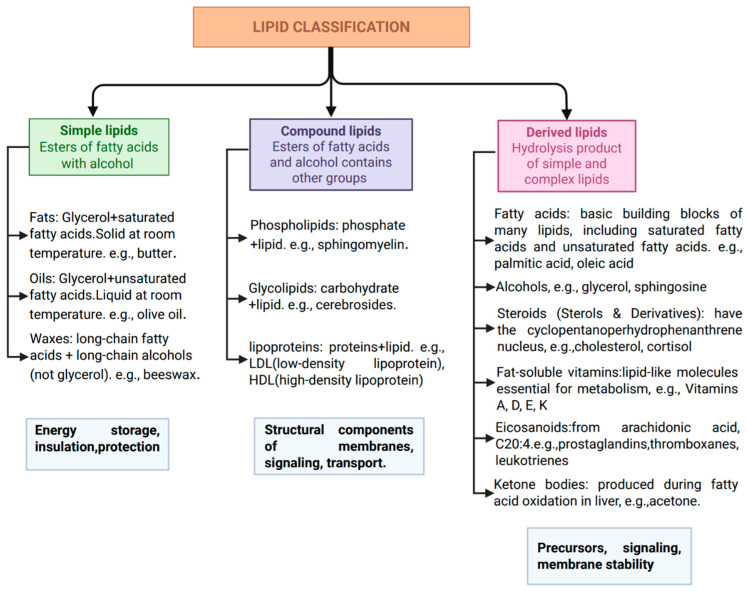
Classification of lipids and their biological functions. Lipids are broadly classified into three categories: simple, compound, and derived lipids. Simple lipids are esters of fatty acids with alcohol and include fats (e.g., butter), oils (e.g., olive oil), and waxes (e.g., beeswax), functioning primarily in energy storage, insulation, and protection. Compound lipids are esters of fatty acids with alcohol and additional groups, including phospholipids, glycolipids, and lipoproteins (e.g., LDL, HDL), and serve as structural components of membranes, as well as mediators of signaling and transport. Derived lipids are products of hydrolysis of simple and compound lipids, including fatty acids, alcohols, steroids (e.g., cholesterol, cortisol), fat-soluble vitamins (A, D, E, K), eicosanoids, and ketone bodies, which act as precursors, signaling molecules, and regulators of membrane stability. Created in BioRender. Cai, Y. (2026) https://BioRender.com/4lyq9j2.

Lipids encompass a wide range of structurally distinct molecules that differ in their biophysical properties and biological roles. As illustrated in Figure 5, they are classified into simple, compound, and derived lipids, each contributing differently to membrane architecture and signaling.

- Cholesterol

Cholesterol is a critical component of the plasma membrane that governs both its structural integrity and functional properties. Experimental studies have demonstrated its influence on lipid-packing density and membrane fluidity [94,95], while molecular dynamics simulations have further revealed its role in regulating lipid tail ordering, membrane curvature [96] and flip-flop behavior [97]. By shaping the physical state of the bilayer, cholesterol thereby modulates ion channel activity, affecting parameters such as open probability [98], unitary conductance [99] and the number of active channels [100]. Cholesterol can also interact directly with ion channels, including Piezo1 and Piezo2. Buyan, Cox [101] and Chong, De Vecchis [102] predicted multiple cholesterol-recognition motifs (termed CRAC and CARC) within Piezo1, specifically 19 CRAC motifs and 39 CARC motifs. Consistently, coarse-grained molecular dynamics simulations of Piezo2 suggested direct cholesterol binding at sites located within the N terminus, potentially contributing to altered channel function [103].

Functional studies support that cholesterol modulates the sensitivity of Piezo1. Ridone, Pandzic [93] reported that cholesterol depletion with methyl-β-cyclodextrin (MβCD) significantly altered Piezo1 channel behavior. In cell-attached recordings, MβCD treatment induced a rightward shift in the Piezo1- Green fluorescent protein (GFP) pressure-response curve in human embryonic kidney (HEK) Piezo1 overexpression system and native Piezo1 neuro 2A (N2A) cells, indicating delayed activation and slower inactivation kinetics. Cholesterol removal also modified the distribution and diffusion of Piezo1-GFP clusters, whereas supplementation with polyunsaturated fatty acids enhances Piezo1 responsiveness to mechanical stimuli. Consistently, cholesterol depletion reduced the amplitude of Yoda1-induced responses in both Piezo1-overexpressing systems and human umbilical vein endothelial cells (HUVECs), as measured using fura-2 assays and outside-out patch-clamp recordings [102]. Piezo1 activity is tightly regulated by cholesterol-rich membrane domains, highlighting the importance of lipid composition in mechanosensitive channel function.

Cholesterol can also modulate Piezo1 activity indirectly through cholesterol-binding scaffolding proteins such as stomatin-like protein 3 (STOML3). Localized within cholesterol-rich lipid rafts, STOML3 regulates membrane mechanics, facilitates force transfer, and thereby enhances the sensitivity of Piezo1 and Piezo2 channels [104]. STOML3 has been shown to lower the mechanical displacement threshold of Piezo channels to the nanometer scale, consistent with native mechanotransduction currents observed in ultrasensitive mechanoreceptors. This effect appears unique to STOML3, as homologous proteins such as STOML1 failed to alter Piezo1 sensitivity. Interestingly, both STOML3 and STOML1 share the ability to prevent desensitization of channel activity following repeated stimulation at a single pilus. Structure–function studies using chimeric constructs have further shown that this modulation effect resides largely within the stomatin domain of STOML3, suggesting that unique features of this domain enable modulation of channel gating. It has also been proposed that STOML3 enhances force transmission by assembling higher-order membrane-associated scaffolds around mechanosensitive channels, with mutations disrupting scaffold formation abolishing its function [105]. Consistent with this model, both cholesterol depletion and STOML3 deficiency similarly attenuate mechanosensitivity in mouse sensory neurons, while in heterologous systems, intact STOML3 is required to preserve membrane mechanics and sensitize Piezo1 and Piezo2 channels [104].

Interestingly, Piezo1 has been shown to influence membrane lipid composition conversely [106]. Transcriptomic analysis of Piezo1 knockout (KO) mouse brains revealed significant downregulation of genes within the cholesterol biosynthesis superpathway of the brain, including Hmgcr, which encodes HMG-CoA reductase, the rate-limiting enzyme of cholesterol synthesis. Consistently, Piezo1 KO neural stem cells (NSCs) exhibited altered lipid composition and reduced free cholesterol levels, while cholesterol supplementation partially rescued their impaired differentiation phenotype in vitro. These findings indicate that Piezo1 contributes to neural development by maintaining intracellular cholesterol homeostasis and suggest a feedback mechanism in which Piezo1 enhances cholesterol biosynthesis.

Recent evidence has linked the regulation of Piezo1 by oxysterols to the development and progression of atherosclerosis. Glogowska, Jose [107] demonstrated that 7-ketocholesterol (7-KC), an oxidized cholesterol metabolite, enhances Piezo1 activation in response to pressure stimulation in both mouse macrophages and transfected HEK cells. Chronic exposure to 7-KC significantly increased Piezo1 current amplitude and prolonged both inactivation and deactivation. These effects were linked to elevated channel expression and to alterations in lipid bilayer properties, as 7-KC incorporation into membranes displaced cholesterol, disrupted lipid packing, and reduced membrane order, thereby facilitating Piezo1 mechanical gating. In contrast, the free form of docosahexaenoic acid (DHA), an atheroprotective lipid, acutely inhibited Piezo1 activity under both control and 7-KC-treated conditions. The opposing actions of 7-KC and DHA indicate that macrophage Piezo1 is differentially regulated by pro- and anti-atherogenic lipids, underscoring its mechanistic role in atherosclerosis and highlighting the channel as a potential therapeutic target in vascular disease.

Kuang, Abrenica [108] demonstrated that cholesterol regulates the responsiveness of Piezo1 to physical confinement and thereby influences cell migration. Cholesterol plays a central role in this process by maintaining lipid order, achieved through intercalation between phospholipids and restriction of membrane fluidity, which is essential for transmitting membrane tension to Piezo1. Through this mechanism, cholesterol enables Piezo1-dependent Ca^2+^ influx, which subsequently activates inverted formin-2 (INF2) to drive actin cytoskeletal remodeling, de-adhesion, and amoeboid (bleb-based) migration in confined environments [109]. Pharmacological inhibition of cholesterol synthesis with Fluvastatin impaired this Ca^2+^ response to confinement, whereas cholesterol supplementation restored it. Furthermore, pharmacological activation of Piezo1 with Yoda1 rescued amoeboid migration in Fluvastatin-treated cells. these findings reveal that lipid regulation of Piezo1 is a critical determinant of confinement sensing and amoeboid migration, thereby linking membrane composition to melanoma cell invasiveness and melanoma progression.

In summary, cholesterol plays a critical role in regulating Piezo channels through multiple mechanisms, including direct binding to the channel and indirect modulation via cholesterol-binding scaffolding proteins. Beyond its biophysical effects on channel activity, cholesterol dependent regulation of Piezo contributes to disease progression, positioning cholesterol Piezo interactions as a promising therapeutic target.

- Phospholipids

Phospholipids are the fundamental structural lipids of cellular membranes, characterized by a hydrophilic head group and two hydrophobic fatty acid tails, which confer amphipathic properties. This molecular organization drives the spontaneous assembly of phospholipids into bilayers, forming the basic framework of biological membranes [110].

Phosphatidylserine (PS) is a negatively charged phospholipid enriched in neural plasma membranes, where it is located in the inner leaflet of the bilayer [111,112]. PS contributes to protein docking sites that are essential for the activation of several signaling pathways [113]. Maintenance of this lipid asymmetry has been shown to be critical for the function of the mechanosensitive ion channel Piezo1 [114]. During myotube formation, PS can become transiently externalized [115], and its subsequent inward translocation by flippases such as ATP11A/CDC50A is required for Piezo1 activation. Loss of flippase activity markedly reduces Piezo1 responsiveness to the chemical agonist Yoda1 without altering plasma membrane tension, indicating that appropriate PS distribution, rather than membrane mechanics alone, is necessary for Piezo1 channel activity. Consistent with this, incorporation of lyso-phosphatidylserine (LysoPS) into the outer leaflet suppressed Piezo1-mediated Ca^2+^ influx in a dose-dependent manner, whereas lyso-phosphatidylcholine (LysoPC) and lyso-phosphatidic acid (LysoPA) had no effect. These findings suggest that phosphoserine headgroups exposed on the extracellular leaflet exert an inhibitory effect on Piezo1 activation [114].

Downstream of this lipid regulation, Piezo1-mediated Ca^2+^ entry activates the RhoA/ROCK pathway, promoting phosphorylation of myosin light chain and assembly of cortical actomyosin fibers. Piezo1 silencing recapitulates the phenotype observed with flippase deficiency, leading to excessive myotube fusion and elongation defects [114], thereby linking lipid asymmetry to cytoskeletal remodeling and muscle development [114,116].

The role of PS in Piezo1 regulation has also been confirmed in red blood cells. In sickle cell anemia (SCA), Yoda1 induces both Ca^2+^ influx and PS externalization, mediated via Piezo1-dependent Ca^2+^ entry and through protein kinase C (PKC) signaling, with PS exposure occurring even in the absence of extracellular Ca^2+^ [117]. It has been proposed that Piezo1 activation may stimulate Ca^2+^-dependent phospholipase C, which in turn modulates PS translocation across the bilayer [118]. Extending this model, recent work identified TMEM16F as the long-sought Ca^2+^-activated phospholipid scramblase (CaPLSase) in red blood cells (RBCs) [119], which is directly activated by Ca^2+^ influx through Piezo1. Importantly, Piezo1–TMEM16F coupling is enhanced in hereditary xerocytosis (HX), a Piezo1 gain-of-function channelopathy, leading to an increased propensity for PS exposure that contributes to anemia, splenomegaly, and thrombosis. Pharmacological inhibition of Piezo1 with agents such as GsMTx-4 or benzbromarone prevents stress-induced PS externalization, echinocytosis, and hemolysis in HX cells [119].

Thus, alterations in PS localization and in regulatory molecules such as flippases, phospholipase C and scramblases, can markedly influence Piezo1 function. Moreover, Yoda1-induced PS externalization may feed back into other PS-dependent signaling cascades, further underscoring the central role of lipid asymmetry in modulating Piezo1 activity and red cell physiology [119].

Phosphatidylinositol 4,5-bisphosphate (PIP2) is a low-abundance but functionally crucial phospholipid located mainly in the inner leaflet of the plasma membrane [120]. PIP2 plays a dual role in cellular signaling, it serves as a substrate for key enzymatic pathways, including phospholipase C (PLC) and phosphoinositide 3-kinase (PI3K), and it functions as a regulatory ligand for a broad spectrum of peripheral and integral membrane proteins [121]. Increasing evidence demonstrates that PIP2 modulates the activity of multiple ion channel families, such as inward rectifier potassium channels (Kir2.1) [122] and transient receptor potential canonical (TRPC) channels [123]. Importantly, PIP2 is also a key regulator of the mechanosensitive Piezo1 channel. Borbiro, Badheka [124] demonstrated that Piezo1 and Piezo2 are phosphoinositide-dependent ion channels, as their currents undergo rapid rundown following patch excision, a phenomenon attributed to the depletion of PI(4,5)P2 and PI(4)P from the excised membrane due to lipid phosphatase activity. Supplementation of excised patches with exogenous PIP2 and its precursor PI(4)P markedly reduced this rundown, highlighting the lipid dependence of Piezo function. These findings suggest that Piezo channels, similar to the mechanosensitive TRAAK and MscS channels [125,126], may also rely on lipid binding, particularly PIP2, to stabilize or facilitate their gating process. PIP2 may act as a cofactor that couples membrane mechanics to Piezo activity, and its depletion disrupts this coupling, thereby impairing mechanosensitivity [127].

To further interrogate PIP2-Piezo1 interactions. Ridone, Vassalli [128] used coarse-grained (CG) molecular dynamics (MD) simulations and showed that, in addition to PIP2, PIP1 and PIP3 are also enriched in the lipid environment surrounding Piezo1. They identified a cluster of four lysine residues (K2166–K2169) near the human Piezo1 pore that is highly conserved across homologues. Loss of this lysine cluster in the Δ4K mutant has been associated with xerocytosis, a hereditary anemia characterized by dehydrated red blood cells [96,129], and electrophysiological recordings revealed that the mutation substantially slows channel deactivation [128]. Building on this, Chong, De Vecchis [102] reported that Piezo1 remodels its local membrane environment by forming a PIP2 annulus through preferential interactions. Computational studies using Piezo1trunc, a truncated construct lacking the flexible N-terminal blade region (approximately the first ~576 amino acids), similarly demonstrated enrichment of phosphoinositides around the channel. In full-length Piezo1 models, numerous PIP2 contacts are observed with the modelled loop and N-terminal residues, suggesting that a PIP2 annulus could create a local signaling hotspot and/or act as a sink that restricts PIP2 availability at distal membrane sites. Jiang, Del Rosario [130] reported an in silico strategy for inducing Piezo1 channel opening by employing all-atom (AA) molecular dynamics simulations of densely assembled Piezo1 clusters with varying levels of membrane-footprint overlap. A 1-Palmitoyl-2-oleoyl-sn-glycero-3-phosphocholine (POPC) bilayer supplemented with PIP2 was simulated for 12 μs to equilibrate the lipid environment and allow the formation of the protein-induced dome. The terminal configuration from this stage was subsequently converted into an all-atom representation and simulated for an additional 2 μs, during which the central pore underwent conformational rearrangements leading to channel opening. Application of a strong electric field in this system produced ion currents that closely matched experimentally observed single-channel conductance and reproduced the effects of conductance-reducing mutations.

A recent paper by Smith, Chuntharpursat-Bon [131] introduced the concept of a “handshake” interaction, which occurs primarily between the peripheral ends of the short helix (SH) and long helix (LH) of adjacent blades of Piezo1. This interaction is mediated by PIP2, which neutralizes positive charges on basic residues, thereby allowing neighboring blades to associate and form a compact hPiezo1 structure. A sufficient pool of PIP2 stabilizes the handshake, whereas a reduction in PIP2 promotes handshake release. PIP2 at the handshake site is fundamental for maintaining a stable interaction. Compact channel conformations generated by stable handshakes are predicted to require greater membrane tension to transition into the flattened, active state. Consistent with these simulation results, both electrophysiological and Ca^2+^ recording studies demonstrated altered channel sensitivity in SH mutants, where putative PIP2-binding residues were disrupted.

Overall, these findings establish PIP2 as both a structural and functional modulator of Piezo1 activity. By directly interacting with conserved residues, stabilizing inter-blade “handshake” contacts, and organizing into an annulus around the channel, PIP2 integrates lipid–protein and protein–protein interactions to fine tune mechanosensitivity. The dynamic interplay between PIP2 availability, Piezo1 conformational states, and membrane mechanics highlights phosphoinositides as key determinants of channel gating. Notably, disease-associated mutations at PIP2-binding sites further highlight its essential physiological role in Piezo1 regulation.

Recent studies indicate that phospholipids can selectively regulate Piezo2 while leaving Piezo1 unaffected. co-expression of TMEM120A (TACAN) suppresses Piezo2 currents without influencing Piezo1 [132], and this effect correlates with elevated levels of phosphatidic acid (PA) and lysophosphatidic acid (LPA) in TMEM120A-expressing cells. Functional studies further demonstrate that intracellular delivery of PA or LPA, as well as long-term exposure to the stable analog carbocyclic PA (ccPA), attenuates Piezo2 activity in both heterologous systems and sensory neurons. Consistently, optogenetic activation of phospholipase D (PLD), the enzyme responsible for PA generation, suppresses Piezo2 function, whereas pharmacological inhibition of PLD enhances Piezo2 activity and increases mechanical sensitivity in vivo [133]. The finding identifies PA and LPA as endogenous lipid regulators that selectively inhibit Piezo2 and highlight PLD signaling as a key modulatory pathway.

- Sphingolipid

Sphingomyelin, a principal component of plasma membranes, plays a pivotal role in regulating cell proliferation and apoptosis [134,135]. Sphingomyelin metabolism has emerged as an important regulator of Piezo1 function in the endothelium [136]. Neutral sphingomyelinase 3 (SMPD3) hydrolyzes sphingomyelin to generate ceramide, a lipid signal shown to be essential for disabling the intrinsic fast inactivation of Piezo1 and thereby enabling sustained mechanosensitive responses in native endothelium. Inhibition or genetic disruption of SMPD3 restores rapid inactivation of Piezo1, while exogenous ceramide reverses this effect and prolongs channel opening during shear stress or continuous flow stimulation. These actions are membrane-delimited and specific to ceramide, as neither sphingomyelin nor phosphocholine alters channel kinetics. Interestingly, sphingomyelin itself influences channel mechanosensitivity by shifting the activation threshold, indicating distinct but complementary lipid-dependent mechanisms: ceramide controls the kinetics of Piezo1 by suppressing inactivation, whereas sphingomyelin modulates its force sensitivity [136].

Consistent with these mechanisms, functional studies in human microvessels demonstrate that inhibition of neutral sphingomyelinase (NSmase) promotes endothelial dysfunction, leading to a switch from nitric oxide (NO)-mediated to hydrogen peroxide (H_2_O_2_)-mediated flow-induced dilation (FID). Notably, Piezo1 agonism with Yoda-1 or supplementation with exogenous ceramide restores NO-dependent FID under NSmase inhibition, while endothelial nitric oxide synthase (eNOS) blockade abolishes this effect, confirming the requirement of Piezo1-dependent signaling for NO production. Parallel experiments in HUVECs show that NSmase inhibition enhances endothelial H_2_O_2_ generation, which is suppressed by either Yoda-1 or ceramide. These findings provide direct evidence that ceramide-induced stabilization of Piezo1 activity sustains NO-mediated endothelial responses and offer mechanistic insight into how ceramide may exert beneficial effects within the human microvasculature [137].

- Fatty Acids

Fatty acids are essential components of cell membranes and cellular lipids which can influence the biophysical properties of cell membranes. Maulucci, Cohen [138] observed that modifications in the fatty acid composition of cell membranes occur in response to changes in temperature, resulting in variations in membrane fluidity. Furthermore, changes in the plasma membrane can influence membrane-associated protein functions and signal transduction pathways, including those of Piezo [139,140].

Romero, Massey [18] demonstrated that fatty acids differentially modulate Piezo1 activity through membrane modelling. The saturated fatty acid margaric acid (MA) increased membrane order and bending stiffness, thereby requiring stronger mechanical stimulation for channel activation and leading to inhibition of Piezo1 currents in a dose-dependent manner. In contrast, polyunsaturated fatty acids (PUFAs) exerted distinct effects on channel inactivation: arachidonic acid and eicosapentaenoic acid reduced the inactivation time of Piezo1, whereas docosahexaenoic acid prolonged it. It has been showed that the regulation of mechanical and fluidic properties of the plasma membrane induced the fatty acid effects on Piezo1 independent of the F-actin structure in the cells. In addition, the difference in Piezo1 properties among the various cell types could be partly due to the variety of lipid profiles in the plasma membranes [18].

In addition to its effects on Piezo1, MA also modulates Piezo2 activity [26]. Specifically, it suppresses Piezo2 function in a dose-dependent manner by elevating the mechanical activation threshold, while leaving the inactivation time constant unchanged. Comparative studies demonstrate greater potency of MA at Piezo1 than Piezo2 [18]. Moreover, disruption of the actin cytoskeleton with latrunculin A increases Piezo2 sensitivity to MA, highlighting the role of cytoskeletal coupling in counteracting membrane-rigidity effects. These mechanisms are further supported by translational studies in human Merkel cell carcinoma cells (MCC13), mouse and rat dorsal root ganglion (DRG) neurons, and human induced pluripotent stem cell (iPSC)-derived neurons, where MA consistently suppresses mechanocurrents and reduces mechanically evoked action potential firing, while no effect on voltage-gated Na^+^ and K^+^ currents. Notably, MA also reverses bradykinin-induced sensitization, restoring mechanocurrents to control level, suggesting a potential role in alleviating inflammation-associated touch hypersensitivity [26].

Recent evidence suggests that dietary PUFAs play an important role in regulating Piezo1 channel activity across different cell types. In particular chondrocytes, both ω3- and ω6-derived PUFAs were attenuated intracellular Ca^2+^ influx in response to mechanical compression and pharmacological activation of Piezo1 [141]. Eicosapentaenoic acid (EPA), DHA, and linoleic acid (LA) were particularly effective in reducing Ca^2+^ responses under compressive stress, while all tested PUFAs similarly suppressed Ca^2+^ signaling induced by the Piezo1 agonist Yoda1. Notably, supplementation with the ω6-PUFA LA reduced membrane stability and promoted lipid droplet accumulation, implying that although LA can dampen Piezo1-mediated Ca^2+^ entry, it may simultaneously increase chondrocyte vulnerability to mechanical injury [141]. Complementary findings from neural models demonstrate that ω3-PUFAs exert anti-inflammatory and neuroprotective effects through the miR-107/Piezo1/NF-κB p65 axis. In lipopolysaccharides (LPS)-induced neuroinflammation, ω3-PUFA supplementation upregulated miR-107 expression, thereby suppressing Piezo1 activity, inhibiting NF-κB signaling, reducing glial activation, and ultimately improving cognitive outcomes [142].

These findings illustrate that fatty acids act as key modulators of Piezo channel activity by altering membrane properties and, in some cases, engaging specific signaling pathways.

### 4.2. MscL and MscS Channels

#### 4.2.1. Overview of MscL and MscS Channels

MscS and MscL are well-characterized mechanosensitive in channels in prokaryotes, firstly discovered in *E. coli* [143]. Both function as emergency release valves to protect cells from lysis under hypoosmotic shock [143]. They are directly gated by membrane tension [144], allowing the rapid efflux of ions and small solutes to prevent cell rupture under hypoosmotic stress [145].

Despite them both playing a role in osmoprotection, the two channels differ substantially in pore size, gating behavior, structure, and physiological function. These two channels reflect distinct families of proteins. The MscS family is highly diverse, with individual organisms encoding multiple homologs that are likely expressed or activated under different environmental conditions [146]. Structurally, MscS is a homoheptamer, each subunit containing three transmembrane helices. It is activated by moderate increases in membrane tension and fine-tunes turgor pressure during the bacterial life cycle [146]. It forms a pore of approximately 13 Å in diameter, which is smaller than that of MscL [147]. A distinctive property of MscS is its ability to adapt to sustained tension: although it responds readily to rapid pressure pulses, gradual application of the same force (e.g., over a 30 s ramp) activates only about half of the channels. Under prolonged subsaturating tension, MscS opens transiently, reflecting two sequential processes: desensitization, characterized by a rightward shift in the activation curve, and inactivation, during which the channel becomes insensitive to tension [146,148].

By contrast, MscL is highly conserved, with only one gene found in any given organism. Structurally, it is a homopentamer in which each subunit contains two transmembrane helices (TM1 and TM2). The TM1 helices form the pore-lining area and TM2 helices are positioned peripherally, interacting with the lipid bilayer to sense membrane tension [149]. Functionally, it opens only under extreme membrane tension, forming a large nonselective pore of ~30 Å in diameter with exceptionally high single-channel conductance (~3 nS) [150]. This emergency release mechanism permits the rapid efflux of ions and small organic molecules, acting as a last-resort “safety valve” that protects cells from catastrophic lysis during severe osmotic downshifts [151].

Together, MscS and MscL establish a tiered protective mechanism: MscS mediates gradual osmolyte efflux under moderate stress, while MscL ensures emergency relief under extreme conditions [152]. The evolutionary conservation of both channels across bacteria and archaea highlights their central role in prokaryotic survival and environmental adaptation [153].

#### 4.2.2. Lipid-Modulation of MscL and MscS Channels

Lipids are key regulators of the mechanosensitive channels MscL and MscS [154,155]. Extensive biophysical and structural studies have demonstrated that membrane composition, curvature, and specific lipid–protein interactions critically influence the activation thresholds of both channels [156,157].

Mechanosensitive channel MscL is sensitive to the physical properties of the surrounding lipid bilayer. Early studies demonstrated that channel gating responds directly to bilayer elasticity and thickness. Incorporation of thinner or curvature-inducing lipids, such as lysophospholipids, lowers the tension threshold for activation by promoting asymmetric leaflet expansion and membrane thinning [158,159]. These findings were reinforced by reconstitution experiments in defined liposomal systems, which showed that enrichment of stiffening lipids, such as cardiolipin or phosphatidylethanolamine (PE) increases bilayer rigidity, thereby elevating the gating threshold and stabilizing the closed state [154].

Beyond global bilayer mechanics, specific lipid–protein interactions also modulate mechanosensitivity. Zhong and Blount [160] demonstrated that the anionic lipid phosphatidylinositol (PI) is essential for gating of the MscL homolog from Mycobacterium tuberculosis (Mt-MscL) in artificial bilayers. When Mt-MscL was incorporated into membranes mimicking the inner membrane of *E. coli*, which lacks PI, no channel activity was observed, highlighting a lipid-specific requirement for channel function. Consistent with this, ion-mobility mass spectrometry revealed that membrane proteins exhibit selective lipid binding, and Mt-MscL displays strong preferential stabilization by PI over other anionic lipids such as phosphatidylglycerol (PG) and cardiolipin (CL) [161].

Computational studies have provided mechanistic insight into these phenomena. Early atomistic simulations revealed that asymmetric lipid composition induces a domed bilayer architecture and promotes persistent interactions between lipid headgroups and specific protein regions, consistent with hydrophobic matching principles [162]. More recent advances introduced the locally distributed tension molecular dynamics (LDT-MD) approach, which applies a localized tension-mimicking bias to annular lipids rather than directly to the protein. This method exploits strong lipid–protein coupling to induce rapid and reversible MscL opening without disrupting membrane integrity, allowing quantitative characterization of structural transitions and the free-energy landscape underlying tension-dependent gating [163]. Vanegas and Arroyo [155] demonstrated that membrane tension generates spatially patterned forces at the protein–lipid interface, mediated by long-lived interactions between positively charged residues and anionic lipids. These findings collectively establish that MscL mechanosensitivity arises from an intricate interplay between bilayer physical properties, localized lipid–protein interactions, and tension-dependent force transmission pathways within the membrane environment.

MscS, in contrast, exhibits both indirect mechanosensitivity, responding to membrane tension and direct lipid regulation at defined binding pockets identified through cryo-EM and simulations [164]. Functional studies comparing gating kinetics in azolectin and pure lipid systems revealed that cardiolipin modulates MscS gating in a dose-dependent manner. In azolectin liposomes, addition of 10% cardiolipin abolishes hysteresis of MscS but leaves MscL largely unaffected, suggesting cardiolipin stabilizes the closed state of MscS. In 1,2-dioleoyl-sn-glycero-3-phosphoethanolamine (DOPE)/1,2-dioleoyl-sn-glycero-3-phosphocholine (DOPC) mixtures, hysteresis is abolished even without cardiolipin, while cardiolipin addition increases opening and closing thresholds for both channels. Notably, MscS gates more frequently in the presence of cardiolipin, destabilizing the open state and potentially influencing bacterial osmotic response [165]. Cryo-EM structures of nanodisc-reconstituted MscS further revealed membrane-interacting regions at the N-terminal end and structurally bound lipids that likely play roles in mechanotransduction, gating, and permeation [164].

These studies show that both MscL and MscS are exquisitely sensitive to their lipid environment. Mechanosensitivity emerges from a combination of bulk bilayer mechanics, specific lipid–protein interactions, and tension-mediated force transmission, highlighting the essential role of membrane lipids in tuning bacterial mechanosensitive channel function.

### 4.3. K2P Channels (TREK-1, TREK-2, and TRAAK)

#### 4.3.1. Overview of K2P Channels

K2P channels constitute a distinct family of K^+^ channels that generate background or “leak” currents, thereby stabilizing the resting membrane potential and controlling cellular excitability [166]. Different from voltage-gated K^+^ channels, K2P channels lack a voltage sensor and are not gated by the membrane potential [167] but instead are constitutively active. The activity of K2P channels can be tuned by diverse chemical and physical factors, which alter the open probability of single K2P channels [166]. To date, 15 subtypes of K2P channels have been identified in mammals, which are classified into 6 subfamilies according to sequence similarity and functional properties [168].

Different K2P channel subtypes exhibit conserved structural and functional characteristics. Each subunit comprises four transmembrane helices and two pore-forming loops, a configuration known as the 4TM/2P architecture [169]. As in other potassium channels, ion selectivity is conferred by the highly conserved selectivity filter motif TxTTxGYGD, whose backbone carbonyls form the binding sites that coordinate permeant K^+^ ions. The C-terminal end of the pore helix does not bind ions directly but provides structural support that maintains the geometry of the filter. This selectivity filter not only establishes high fidelity for K^+^ conduction but also constitutes a critical site of channel gating, where conformational rearrangements regulate transitions between the open and closed states [170].

Importantly, beyond their shared architecture, several K2P subunits TREK-1, TREK-2, and TRAAK display inherent mechanosensitivity [34], a property not common to all members of the K2P family [171]. TREK and TRAAK channels can be activated by a variety of mechanical stimuli, including membrane stretch, poking, osmotic swelling, and fluid jet stimulation [34]. They respond across a wide range of membrane tensions, from ~0.5 mN/m to ~12 mN/m, with open probability scaling proportionally to the applied tension until the bilayer ruptures [171]. In addition to mechanosensitivity, these channels exhibit polymodal regulation by lipids, PUFAs, temperature, and pH [172].

TREK-1, TREK-2 and TRAAK channels play pivotal roles in regulating neuronal excitability and sensory processing. Both channels are expressed in the central and peripheral nervous systems, including sensory neurons of the dorsal root ganglia, where other mechanosensitive cation-permeable channels are also present [172]. Although widely expressed in sensory neurons, TREK and TRAAK channels do not directly generate action potentials; rather, they conduct hyperpolarizing K^+^ currents. In systems where mechanical stimulation also activates depolarizing ion channels such as Piezo1, TRAAK-mediated hyperpolarization can counteract excitatory currents, thereby contributing to stabilization of the membrane potential [46]. Consistently, mice lacking both TREK-2 and TRAAK display enhanced sensitivity to mechanical stimuli, as evidenced by increased responses to von Frey filament application and paw injection of hypotonic solution [173,174].

Pathophysiologically, dysfunction of TREK/TRAAK channels has been implicated in a range of neurological and psychiatric disorders. Altered TREK-1 activity has been associated with depression, as TREK-1 knockout mice display resistance to depression-like behaviors [175], sparking interest in TREK-1 inhibitors as potential antidepressant therapies. Abnormal K2P channel activity has also been linked to epilepsy, migraine, and other conditions characterized by altered neuronal excitability [176]. Notably, a de novo gain-of-function missense mutation in the KCNK4 gene, which encodes the TRAAK channel, has been reported to cause a recognizable neurodevelopmental syndrome characterized by facial dysmorphism, hypertrichosis, epilepsy, intellectual disability/developmental delay, and gingival overgrowth [177]. TREK-1, TREK-2, and TRAAK channels act as key modulators of neuronal excitability and sensory function, integrating mechanical and chemical signals to maintain cellular homeostasis.

#### 4.3.2. Lipid Modulation of K2P Channels

Lipids play a central role in the regulation of K2P mechanosensitive channels, particularly the TREK and TRAAK subfamily. TREK-1 and TRAAK, both outwardly rectifying K2P channels, are activated by mechanical stimuli such as membrane stretch, cell swelling, and shear stress, but exhibit negligible basal activity at atmospheric pressure [173]. Convex curvature of the plasma membrane can effectively gate these channels and mechanosensitivity persists even when the cytoskeleton is disrupted, indicating that the activating force arises directly from the lipid bilayer [172]. Consistent with this FFL model, polyunsaturated fatty acids such as arachidonic acid, amphipathic anions like trinitrophenol, and signaling lipids including lysophospholipids and platelet-activating factor mimic mechanical stress and act as potent activators [178]. Reconstitution experiments provided direct evidence that mechanosensitivity is an intrinsic property of TREK-1 and TRAAK: when purified proteins were incorporated into artificial bilayers devoid of cellular components, they retained robust and symmetric activation by both positive and negative pressures, confirming that gating is driven by lateral membrane tension [46].

Structural and computational analyses extended these findings to TREK-2, demonstrating that bilayer stretch promotes a rapid expansion from the “down” to the “up” state, with reduced lipid density around the protein facilitating conformational switching. Importantly, gating does not involve lipid occlusion of the pore, but rather a bilayer-mediated coupling between membrane mechanics and selectivity filter ion occupancy [179].

Specific phospholipids also exert powerful modulatory effects on TREK channels. Early studies identified phosphatidylethanolamine, phosphatidylinositol, phosphatidylserine, phosphatidic acid (PA), and PIP2 as activators of TREK-1, with PA being the most potent, and mapped a cluster of basic residues in the proximal C-terminus adjacent to TM4 as a potential PIP2-binding site [180]. Negatively charged lipids were proposed to interact electrostatically with these residues to facilitate channel opening, although subsequent work revealed that PIP2 can also inhibit TREK-1, suggesting dual and context-dependent effects [181,182,183]. Additional binding regions in the distal C-terminus have been proposed [184], and direct lipid interactions have been confirmed in vitro for TREK-1 [183] and TRAAK [185]. Cryo-EM structures revealed that PA can insert behind the selectivity filter to promote conformational changes that favor channel opening, while PA and phosphatidylethanolamine compete at the cytoplasmic face to regulate TM4 helix motions, demonstrating distinct lipid-dependent gating pathways [186]. Recent native mass spectrometry studies further showed that TREK-2 preferentially binds anionic lipids such as PA, PG, and PIP2 over zwitterionic lipids like PE and PC, with multiple high-affinity sites consistent with electropositive patches in the C-terminal domain that stabilize open conformations [187].

Taken together, these findings establish lipids as versatile and multifaceted regulators of K2P mechanosensitive channels, acting both as direct force mediators of bilayer tension and as site-specific chemical modulators that shape channel gating through dynamic interactions with the TM4 helix and selectivity filter.

### 4.4. TRP Family

#### 4.4.1. Overview of TRP Family

Transient receptor potential (TRP) channels comprise a large and diverse superfamily of nonselective cation-permeable ion channels that play a critical role in sensory physiology and cellular signaling [188]. The family was first identified in the fruit fly Drosophila melanogaster, where a mutant fly displayed a transient receptor potential (TRP) phenotype in response to continuous light stimulation, in contrast to the sustained electroretinogram recorded in wild-type flies [189]. The gene responsible for this phenotype was subsequently cloned and characterized by Craig Montell and Gerald Rubin in 1989 [190].

TRP channels are now recognized as polymodal detectors activated by a broad range of physical and chemical stimuli, including temperature, pH, osmotic stress, and mechanical forces [191]. Structurally, TRP channels share a conserved architecture of six transmembrane helices (S1–S6), in which the S1–S4 segments form peripheral sensing domains, while the pore-forming loop resides between S5 and S6 [192,193].

In mammals, 28 TRP channel subtypes have been identified and are classified into six major subfamilies based on sequence homology: TRPC (canonical), TRPV (vanilloid), TRPM (melastatin), TRPA (ankyrin), TRPP (polycystin), and TRPML (mucolipin) [1,194]. Among these, several members, most notably TRPC1, TRPC6, TRPV2, TRPV4, TRPA1, and TRPP2 have been involved in mechanotransduction, mediating cellular responses to membrane stretch, shear stress, and osmotic changes [195].

In addition to the six mammalian TRP subfamilies, the TRPN(NompC) subfamily is present in invertebrates and lower vertebrates but absent in mammals [196]. The Drosophila melanogaster No mechanoreceptor potential C (NompC) channel represents one of the earliest genetically identified mechanotransduction channels [197]. NompC contains an unusually long N-terminal region composed of approximately 29 ankyrin repeats, which are thought to function as a cytoskeletal tether [196]. These ankyrin repeats have been proposed to act as a gating spring, transmitting mechanical forces from the cytoskeleton directly to the channel pore in a force-from-filament manner [198,199]. Functionally, NompC is essential for touch sensation, proprioception, and locomotor coordination in Drosophila, and it has provided fundamental insights into tethered models of TRP channel mechanogating [200].

Mechanosensitive TRP channels act as polymodal integrators of mechanical and chemical cues, thereby enabling adaptive responses across diverse tissues and organ systems. In the vasculature, TRPV4 channels expressed in endothelial and vascular smooth muscle cells play a critical role in the regulation of vascular tone. Activation of TRPV4 induces localized increases in intracellular Ca^2+^, which promote endothelium-dependent vasodilation [9,201]. The broad spectrum of stimuli capable of activating TRPV4, combined with its strategic localization in endothelial cells, facilitates flow- and shear stress–mediated release of endothelium-derived hyperpolarizing factors (EDHF), thereby contributing to vascular relaxation and homeostatic control of blood flow [202]. TRPC6 plays a crucial role in vascular mechanosensation; normally, increased intraluminal pressure evokes myogenic vasoconstriction (the Bayliss effect) through stretch-activated cation channels in vascular smooth muscle cells (VSMCs) [203]. TRPC6 deficiency attenuates pressure-induced depolarization and contraction, impairing myogenic tone. TRPC6-null mice display elevated basal blood pressure, enhanced agonist-induced arterial contractility, increased basal cation influx, and more depolarized VSMCs, underscoring its essential role in pressure-dependent vascular regulation [204].

In the nervous system, TRPA1 contributes to mechanonociception and somatosensory processing, mediating responses to noxious mechanical stimuli and promoting pain hypersensitivity [205]. In the kidney, TRPP2 (polycystin-2) functions in complex with polycystin-1 as a ciliary mechanosensor that detects tubular fluid flow and triggers Ca^2+^ influx, which is essential for renal epithelial homeostasis [206].

Disruption of mechanosensitive TRP channel function has been linked to a range of human diseases. Mutations in TRPP2 cause autosomal dominant polycystic kidney disease (ADPKD) [207]. Dysregulated TRPC6 activity has been associated with focal segmental glomerulosclerosis (FSGS), as well as with pulmonary hypertension due to its role in smooth muscle contraction and vascular remodeling [208]. Gain-of-function mutations in TRPV4 underlie a wide spectrum of channelopathies, including skeletal dysplasias, hereditary motor and sensory neuropathies, and Charcot-Marie-Tooth-like syndromes [209]. Hyperactivation of TRPA1 contributes to chronic pain and inflammatory hypersensitivity [210].

#### 4.4.2. Lipid Regulation of TRP Family

A wide range of endogenous lipids has been identified as modulators of TRP channels. These include metabolites of PLC and phospholipase A_2_ (PLA_2_), omega-3 (ω-3) and omega-6 (ω-6) PUFAs, oxidized lipids, sphingolipids, cholesterol and related steroids, as well as intermediates of the mevalonate pathway [211]. These molecules regulate TRP channel activity through two main mechanisms: Either by directly binding to specific channel domains or by altering the mechanical properties of the surrounding lipid bilayer, thereby shaping the FFL model of mechanosensitive gating [212].

- Cholesterol

Within the TRPC subfamily, cholesterol-rich microdomains play a central role in mechanosensitivity. TRPC1 has been linked to lipid rafts and caveolae, and its association with caveolin-1 appears essential for proper function [213,214]. Depletion of cholesterol with methyl-β-cyclodextrin suppresses store-operated Ca^2+^ entry and TRPC1-dependent currents, while cholesterol enrichment facilitates these processes [215]. Similarly, cholesterol loading of cells was found to have a positive effect on signals relating to TRPC3 [216,217]. TRPC6 represents a canonical example of lipid-gated mechanosensitivity: it is directly activated by the PLC-derived second messenger diacylglycerol (DAG) in a membrane-delimited manner, with mechanical stretch amplifying this effect [218,219]. Additionally, the cholesterol-binding protein podocin enhances TRPC6 activity in a cholesterol-dependent manner, particularly at the slit diaphragm of podocytes, illustrating the dual role of specific lipid ligands and membrane organization in tuning mechanosensory signaling [220,221].

TRPV channels are also tightly controlled by lipid interactions, especially with cholesterol. Increased cholesterol content consistently suppresses TRPV4 currents in diverse cell types, including endothelial cells [222], bone cells [223], Müller glia [224], and Xenopus oocytes, whereas cholesterol depletion enhances channel activity and Ca^2+^ influx [225]. Cholesterol has been shown to inhibit TRPV4 responses to both chemical agonists and shear stress in mouse mesenteric arteries and in human coronary artery endothelial cells [9].

Evidence indicates that TRPV channels, particularly TRPV1 and TRPV4, are regulated through direct interactions with cholesterol. Early work on TRPV1 demonstrated that cholesterol binds directly to the channel and inhibits its function [226]. Cholesterol was shown to associate with a conserved cholesterol-recognition amino acid consensus (CARC) motif. Mutations in this domain abolished the inhibitory effect of cholesterol on TRPV1, confirming the functional importance of this binding site [226]. Subsequent studies revealed that cholesterol also associates with a conserved CARC motif located in the TM4-loop-TM5 region of TRPV4 [223,227]. The loop 4 domain alone is capable of interacting not only with cholesterol but also with its biosynthetic precursor mevalonate and other sterol derivatives such as stigmasterol and aldosterone. Consistent with this, the lateral mobility of TRPV4 fused to GFP has been shown to vary in accordance with membrane cholesterol levels. Interestingly, the evolutionary trajectory of TRPV4 displays striking parallels with the cholesterol biosynthetic pathway at the genetic, molecular, and metabolic levels, suggesting a co-adaptive relationship between channel function and sterol metabolism [227]. Structural modeling further indicated that cholesterol may occupy the binding pocket in multiple orientations. Crucially, mutation of a key residue within this motif (R616Q) abolishes cholesterol sensitivity, demonstrating that this site is indispensable for cholesterol-dependent inhibition of TRPV4 activity [223].

Cholesterol binding also appears to influence the membrane localization and molecular interactions of TRPV4. Wild-type TRPV4 co-localizes with caveolin-1 and partitions into caveolae, whereas the R616Q mutant fails to do so, suggesting that cholesterol binding is required for caveolar targeting [223]. Interestingly, the association of TRPV4 with caveolae is not consistent across cell types. While bone and mesenchymal stem cells exhibit caveolin-1 co-localization [223], trabecular meshwork epithelial cells and Xenopus oocytes do not [222]. Nevertheless, cholesterol sensitivity of the channel is preserved in these contexts: enrichment with cholesterol suppresses TRPV4 activity, whereas depletion enhances it [222]. This indicates that direct cholesterol regulation of TRPV4 is not strictly dependent on caveolar partitioning.

- Phospholipids

The regulatory influence of PIP2 on TRP channels is highly variable across subfamilies and even among individual members [228,229]. In TRPV2, PIP2 promotes channel activation, and subsequent reductions in membrane PIP2 during channel opening contribute substantially to Ca^2+^-dependent desensitization [230]. TRPV4, a polymodal channel involved in sensing temperature, osmotic stress, and mechanical force, exhibits a more complex mode of regulation. Single-channel studies demonstrate that PIP2 acts as a cofactor for thermo-activation, requiring interaction with a cluster of positively charged residues in the N-terminal region preceding the proline-rich domain (residues 132–144). In excised patches, loss of PIP2 abolishes heat responsiveness, while exogenous PIP2 restores channel activity. Disruption of this interaction also enhances fluorescence resonance energy transfer (FRET) between cytosolic domains of TRPV4, suggesting a PIP2-dependent conformational rearrangement. [231]. Conversely, whole-cell recordings indicate that PIP2 can suppress TRPV4 activity through binding of its inositol headgroup to the ankyrin repeat domain (ARD) [232]. Consistent with this inhibitory role, TRPV4 channels in capillary endothelial cells display extremely low basal activity due to tonic PIP2-mediated suppression, which is relieved by PIP2 depletion independently of its hydrolysis products DAG and IP_3_ [229,233].

Phospholipids also play an essential structural role in channel activation. Structural and functional analyses of the Drosophila mechanotransduction channel NompC demonstrate that lipid–protein interactions are critical for mechanogating [199,234]. Cryo-EM structures revealed a well-defined lipid density in the intracellular leaflet occupying a hydrophobic cleft formed by the pre-S1 elbow, S1, S4, the S4–S5 linker, and S5 of an adjacent subunit [199]. The lipid headgroup is coordinated by His1423 within the S4–S5 linker, a key gating element. Mutation of His1423 abolishes mechanosensitivity without affecting membrane expression, whereas mutation of the adjacent His1424 has no functional effect [199], indicating that this specific lipid interaction is required for channel activation.

Although modeled as a phospholipid, the precise lipid species was not identified. The persistence of lipid density in amphipol-reconstituted structures suggests tight, likely endogenous binding [199]. Molecular dynamics simulations further support this mechanism, showing that phosphatidylcholine species such as POPC can occupy the same cleft, interact with His1423, and stabilize the S4–S5 linker conformation [234]. A recent study demonstrated that mechanical stimulation drives a rotational conformational change within the transmembrane region. The identified coupling between the TRP domain and the S4–S5 linker indicates that these elements likely operate as an integrated amphipathic module, contributing to the detection of mechanical force and the transmission of gating signals to the pore [235]. The findings support a model in which tightly bound phospholipid interactions and twist-based conformational coupling enable efficient mechanical force transduction in NompC.

- Fatty acids

Fatty acids have been shown to regulate TRPV4 channels through multiple mechanisms involving both membrane composition and metabolic signaling. Caires, Sierra-Valdez [27] proposed that ω-3 PUFAs modulate endothelial cell reactivity by altering membrane biophysical properties, thereby controlling the number of TRPV4 channels available for activation and attenuating Ca^2+^-dependent desensitization. *C. elegans* TRPV4 mutants lacking free long-chain PUFAs or those incorporated into phospholipids fail to respond to chemical and physical stimuli, while supplementation with the epoxygenated metabolite 17,18-epoxyeicosatetraenoic acid (17,18-EEQ) enhances TRPV4 activity. Similarly, human microvascular endothelial cell (HMVEC) membranes enriched with ω-3 PUFAs exhibit increased fluidity and reduced bending stiffness, reinforcing the link between membrane composition and TRPV4 function [27]. Beyond these structural effects, metabolic regulation has also been described [236], in pulmonary arterial hypertension (PAH), microvascular endothelial cells (MVECs) with elevated intracellular lipid content show increased TRPV4 activity, whereas inhibition of fatty acid oxidation restores basal calcium levels. The metabolite β-hydroxybutyrate (BOHB), a product of fatty acid oxidation, was identified as a key mediator, with exogenous BOHB alone sufficient to sensitize TRPV4 channels in rat and mouse MVECs. A transpulmonary BOHB gradient has been detected in PAH patients, suggesting that altered lipid metabolism and metabolite-driven sensitization collectively contribute to pathological TRPV4 activation [236].

### 4.5. OSCA/TMEM63 Channels

#### 4.5.1. Overview of OSCA/TMEM63 Channels

The OSCA/TMEM63 family represents a recently identified class of multi-pass, inherently mechanosensitive ion channels [237]. These proteins were first characterized in *Arabidopsis thaliana*, where mutations in OSCA1.1 impaired rapid osmotic stress-induced Ca^2+^ elevation, suggesting a role of OSCAs as osmosensors in plants [238]. The OSCA family in plants consists of 15 paralogs [238]. Their mammalian homologs, known as TMEM63A, TMEM63B, and TMEM63C, were subsequently demonstrated to function as mechanically activated cation channels, establishing OSCA/TMEM63 proteins as evolutionarily conserved mechanosensitive channels across eukaryotes [239].

Electrophysiological evidence confirmed that OSCAs are bona fide pore-forming mechanosensitive ion channels [239]. Cryo-EM revealed that OSCA/TMEM63 proteins assemble as large homodimeric complexes, with each subunit comprising 11 transmembrane helices (TM0-TM10) and an intracellular domain (ICD) [240]. The ICD is formed primarily by a long TM2–TM3 intracellular linker (IL2) and a C-terminal tail. Notable structural features include (i) a hook-shaped loop within IL2 that penetrates the lipid bilayer, and (ii) a horizontal amphipathic helix (IL1H1) that perturbs the lower leaflet of the membrane in molecular dynamics simulations, both proposed to act as membrane tension sensors. The pore region is located between TM6 and TM7, with a hydrophilic cavity that serves as the ion permeation pathway [240]. Functionally, OSCA/TMEM63 proteins form nonselective cation channels permeable to Ca^2+^, Na^+^, and K^+^ [241].

Although the study of mammalian TMEM63 channels remains at an early stage, accumulating evidence points to their potential involvement in disease. TMEM63A heterozygous variants have been identified in patients with infantile-onset transient hypomyelination; affected individuals showed delayed but ultimately favorable myelination and developmental progress [242]. Ten distinct heterozygous de novo variants of TMEM63B in 17 unrelated individuals with early-onset developmental and epileptic encephalopathy (DEE), all associated with white matter disease, corpus callosum abnormalities, and variable cortical, cerebellar, and hematological abnormalities [243]. In mice, TMEM63B deficiency results in deafness, suggesting a role as an osmosensor in outer hair cells of the inner ear [244]. TMEM63C, localized to the endoplasmic reticulum (ER) and enriched at mitochondria–ER contact sites (MERCs), has been implicated in organelle homeostasis, as its knockdown induces mitochondrial and ER morphological defects [245].

OSCA/TMEM63 proteins define an evolutionarily conserved family of mechanosensitive ion channels, with essential roles in osmosensation, cellular signaling, and organelle function. However, despite recent advances in structural biology, the precise mechano-gating mechanisms of the TMEM63 family remain to be explored.

#### 4.5.2. Lipid Regulation of OSCA/TMEM63 Channels

As a recently identified family of mechanosensitive ion channels conserved across eukaryotes [239], OSCA/TMEM63 channels have only a few lipid modulators; however, growing evidence indicates that the lipid environment plays a central role in their regulation.

Jojoa-Cruz, Saotome [240] revealed a dimeric assembly with eleven transmembrane helices per subunit that closely resembles the architecture of TMEM16 proteins. Molecular dynamics simulations further showed that lipids can occlude the cytoplasmic pore and interact via phosphate headgroups with lysine residues on TM4 and TM6b, implicating lipid binding directly in channel gating. Extending these observations, Zhang, Shan [237] determined cryo-EM structures of mammalian TMEM63A and plant OSCA1.1 and OSCA3.1 and emphasized the integral role of bound lipids in OSCA/TMEM63 mechanosensation. Their analysis demonstrated that the dimer interface forms a central cavity accommodating modulating lipids, which couple the two subunits and fine-tune channel sensitivity to membrane tension, while a cytosolic plug lipid occludes the pore to prevent ion conduction in the closed state. These studies suggest that OSCA/TMEM63 channels utilize a gating strategy that integrates lipid-mediated control with features reminiscent of calcium-dependent gating in the TMEM16 family.

Recent structural and functional studies have revealed that OSCA/TMEM63 channels employ lipid-dependent mechanisms for both ion conduction and membrane regulation [246]. Cryo-EM analyses revealed that gating involves coordinated pore dilation, subunit rearrangement, and lipid displacement, with conical lipids such as lyso-phosphatidylcholine facilitating channel opening. In addition, TMEM63B functions as a membrane structure–responsive lipid scramblase that redistributes phospholipids in response to changes in bilayer mechanics, with disease-linked mutations disrupting lipid asymmetry [246]. Consistent with this, combined approaches including MD simulations, cryo-EM as well as in vitro and in vivo scramblase assays, demonstrated that OSCA1.1, OSCA1.2, OSCA2.2, and TMEM63A/B can mediate lipid translocation. This scrambling activity is inhibited by cholesterol but can be activated in wild-type proteins by mechanical forces that induce morphological changes in membranes. At physiological expression levels, lipid scrambling appears to protect membranes against excessive mechanical stress, suggesting a broader role for OSCA/TMEM63 proteins in maintaining membrane mechano-resilience [247].

Lipids show the potential to be involved in OSCA/TMEM63 channel gating, functioning both as structural components and as regulators of mechanosensitivity.

### 4.6. MET Channel

MET channels are specialized mechanosensitive ion channels located in the stereociliary bundles of inner ear hair cells [248], where they serve as the molecular gatekeepers of hearing and balance. By converting mechanical stimuli, such as sound-induced vibrations or head movements, into electrical signals, MET channels initiate receptor potentials and subsequent synaptic transmission to auditory and vestibular afferents [249]. Channel opening occurs within microseconds following stereocilia deflection, allowing rapid cation influx that underlies mechanotransduction [250].

The pore-forming subunits of MET channels are encoded by transmembrane channel-like proteins (TMC1 and TMC2) [251]. Proper assembly and function require several accessory proteins. TMIE (transmembrane inner ear protein) and LHFPL5 (known as tetraspan membrane protein of hair cell stereocilia (TMHS) are essential for channel targeting, gating, and stabilization [252]. TMIE, identified through human deafness gene DFNB6 and the spinner mouse model, directly binds TMC1/2, and disruption of this interaction abolishes mechanotransduction [253,254]. Moreover, TMIE interacts with LHFPL5 and PCDH15, anchoring the channel complex to the lower end of tip links. CIB2 and CIB3, members of the calcium- and integrin-binding protein family, provide further regulatory input. These small EF-hand proteins facilitate Ca^2+^ binding and interact with TMC1/2 to stabilize MET channel function [255]. Genetic mutations in CIB2 are associated with nonsyndromic deafness (DFNB48) and Usher syndrome type 1J, with knockout or point mutations causing impaired mechanotransduction and progressive hair bundle degeneration [256,257].

Although structural and biochemical studies have advanced understanding of the MET complex, high-resolution cryo-EM structures of the full-length channel remain to be resolved.

### 4.7. DEG/ENaC and ASIC Channels

#### 4.7.1. Overview of DEG/ENaC and ASIC Channels

The DEG/ENaC superfamily comprises a group of voltage-independent, sodium-selective ion channels broadly expressed across the animal kingdom, where they regulate diverse physiological processes such as mechanosensation, pain perception, and sodium homeostasis [258]. Members of this superfamily include four distinct channel classes: (i) ENaCs, which play a central role in sodium reabsorption and systemic salt balance [259]; (ii) acid-sensing ion channels (ASICs), proton-gated cation channels that mediate neuronal excitability and nociceptive signaling [260]; (iii) DEGs, first identified in Caenorhabditis elegans and implicated in mechanosensation [261]; and (iv) FMRF-amide-gated sodium channels (FaNaCs), neuropeptide-gated channels originally described in invertebrates [262].

All DEG/ENaC channels share a similar trimeric structure, consisting of two transmembrane helices(TM1, TM2), a large ectodomain, and intracellular N- and C-terminal domains [263]. The extracellular domain, with its characteristic “hand-like” structure, interacts with various stimuli that modulate channel activity, while TM2 helices from three subunits line the ion conduction pore [264]. Channels are typically sodium-selective but exhibit variable permeability to other cations depending on subtype and gating context [265].

In mammals, ASICs represent the most prominent subgroup of the family [266]. While classically defined as proton-gated channels activated by extracellular acidification, ASICs have also contributed to mechanotransduction in peripheral sensory neurons [267]. ASIC2 is abundantly expressed in cutaneous mechanoreceptors, including hair follicle/vibrissal afferents, penicillate endings, intraepidermal nerve endings, Merkel cells, and Meissner corpuscles, where it contributes to distinct sensory modalities [268]. Functional studies demonstrated that ASIC2 knockout mice exhibit reduced sensitivity of both rapidly adapting and slowly adapting mechanoreceptors [269]. However, contrasting evidence suggests that cutaneous mechanosensation may remain intact in ASIC2-deficient mice [270].

ASICs are involved in diverse physiological processes, including synaptic transmission [271], plasticity [272], learning, memory, and fear responses [273]. Beyond their physiological roles, ASICs have also been implicated in multiple pathological conditions, including chronic pain [274], Parkinson’s disease, multiple sclerosis, Huntington’s disease [275], and ischemic [276].

#### 4.7.2. Lipid Regulation of DEG/ENaC and ASIC Channels

Lipid modulation is an important regulatory mechanism for members of the DEG/ENaC/ASIC family of ion channels. PUFAs such as arachidonic acid (AA) and DHA enhance ASIC currents, with AA shown to potentiate ASIC1a and ASIC3 by shifting the pH dependence of activation, thereby increasing proton-evoked currents and neuronal excitability in dorsal root ganglion neurons [277,278]. Consistent with a physiological role, joint exudates from patients with inflammation, containing lysophosphatidylcholine (LPC) and AA, can activate ASICs even in the absence of extracellular acidification [279]. More recently, structural and functional analyses revealed that both PUFAs and their derivatives regulate ASIC3 by altering multiple biophysical properties of the channel, with efficacy determined by features of both the hydrophobic tail and polar head group [280]. While tail length and unsaturation influence the strength of modulation, the head group is a critical determinant, with more negatively charged substitutions producing greater potentiation, and in some cases directly activating channels at neutral pH. These findings also identified a putative PUFA interaction site on the first transmembrane helix near the outer membrane leaflet, supporting a model in which specific lipid–protein interactions shape ASIC gating and extend the role of lipids from passive membrane components to active modulators of sensory ion channel function [280].

Cholesterol has been shown to stabilize ENaC within ordered membrane domains, and its depletion decreases channel activity, underscoring the importance of membrane composition and organization in ENaC regulation. Using patch-clamp electrophysiology combined with confocal microscopy, Zhai, Liu [281] demonstrated that ENaC and cholesterol are co-localized with PIP2 in microvilli, and that cholesterol depletion reduces both ENaC surface density and activity by lowering local PIP2 levels. More recently, Ríos-Medina, Rico-Chávez [282] provided a broader lipidomic perspective, analyzing whole-cell lipid profiles to explore the relationship between structural lipids of the plasma membrane and protein machinery controlling ENaC retrieval and degradation in neutrophils. Although this study did not specifically isolate plasma membrane lipids, it emphasizes that physiological and pathological changes in lipid composition are most pronounced at the cell surface, reinforcing the view that ENaC function is intimately linked to the dynamic lipid environment of the plasma membrane.

**Figure 6:**
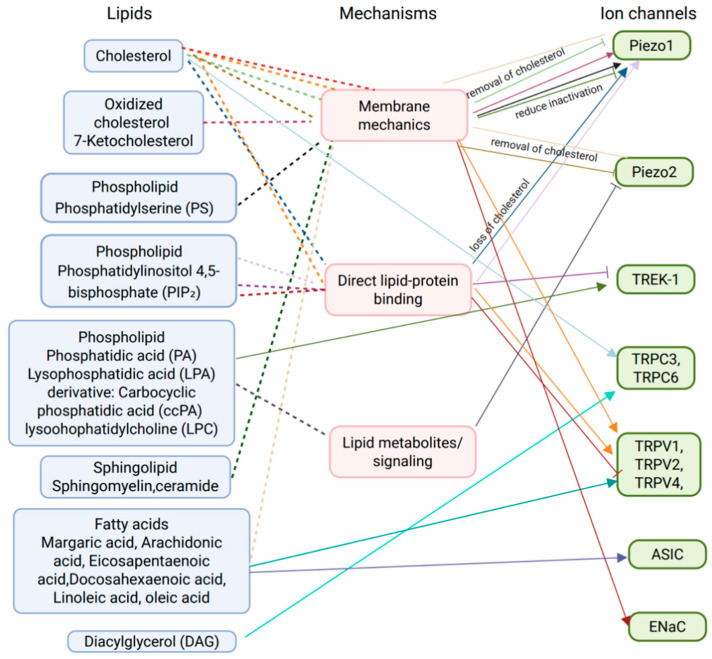
Mechanisms by which lipids regulate mechanosensitive ion channels. Schematic representation of lipid classes, their modes of action, and associated ion channels. Lipids influence channel function through three main mechanisms: (i) modulation of membrane mechanics, (ii) direct lipid–protein interactions, and (iii) lipid metabolites/signaling pathways. These processes regulate the activity of mechanosensitive channels, including Piezo1, Piezo2, TREK-1, TRPC3/6, TRPV1/2/4, ASIC, and ENaC. Created in BioRender. Cai, Y. (2026) https://BioRender.com/4lyq9j2.

The regulatory effects of lipids on mechanosensitive ion channels discussed in this section are summarized in Table 2 and Figure 6, including the lipid species, target ion channels, effects on channel activity, underlying mechanisms, experimental models, and references.

## 5. Therapeutic Potential of Mechanosensing Modifications by Lipids

Lipid-mediated regulation of mechanosensitive ion channels constitutes a pivotal interface between cellular metabolism, membrane biophysics, and ion channel physiology. By translating environmental cues and metabolic states into channel activity, lipids not only refine mechanotransduction under physiological conditions but also influence pathological adaptations. This lipid–channel axis, therefore, represents a promising target for novel therapeutic interventions in regenerative medicine and disease.

### 5.1. Lipid Regulation of Endothelial Mechanotransduction in Neurodegeneration

The mechanotransductive properties of glial cells are increasingly recognized as important contributors to neurodegenerative diseases. Ivkovic, Major [284] highlighted the capacity of fatty acids to modulate Piezo1-dependent mechanosensitivity in glial cells, with implications for Alzheimer’s disease (AD). Supplementation with PUFAs has been shown to alter microglial polarization, enhance amyloid plaque clearance, and reshape immune responses. Given that Piezo1 activity is involved in similar processes of microglial regulation, these observations suggest new strategies to leverage microglia in AD management and therapy [284].

### 5.2. Lipid Regulation of Joint Pathology

Mechanosensitive channels also participate in joint pathology [141]. In osteoarthritis, ω3-PUFAs exert protective effects on cartilage by reducing Piezo1/TRPV4-mediated mechanosignaling, stabilizing membrane biophysical properties, and attenuating pro-inflammatory signaling. In contrast, ω6-PUFAs appear to compromise membrane integrity and potentially exacerbate cartilage degeneration. Thus, optimizing the optimal balance of PUFA intake remains an important avenue for nutritional strategies aimed at osteoarthritis prevention and treatment [141].

### 5.3. Lipid Regulation of Mechanotransduction in Sickle Cell Disease (SCD)

The therapeutic relevance of lipid–channel interactions is further underscored in SCD, a disorder associated with gain-of-function mutations in Piezo1 [285]. Enhanced channel activity in sickle erythrocytes has been observed across both human patients and murine models, contributing to abnormal cation permeability and hemolysis. Dietary enrichment with EPA restored Piezo1 function in SCD mice, resulting in reduced plasma hemoglobin and indirect bilirubin levels alongside lowered systemic inflammation. These findings establish Piezo1 as a critical contributor to SCD pathophysiology and highlight the potential of dietary lipid modulation as a therapeutic approach for hematological disorders [286].

### 5.4. Lipid Regulation of Endothelial Mechanotransduction in Cardiovascular Disease

From a vascular perspective, cholesterol–channel interactions provide additional layers of regulation. Alterations in membrane cholesterol modulate the activity of mechanosensitive channels such as TRPV4, thereby influencing vasodilatory responses. In endothelial cells, chronic hypoxia (CH) induces a marked reduction in membrane cholesterol, which enhances TRPV4-dependent Ca^2+^ entry and promotes activation of large-conductance Ca^2+^-activated potassium (BK) channels. Proximity ligation assays revealed close spatial organization of caveolin-1 (Cav-1), TRPV4, and BK channels, underscoring the importance of cholesterol-rich caveolar microdomains in assembling and stabilizing this signaling unit. Disruption of caveolae through cholesterol depletion with methyl-β-cyclodextrin significantly attenuated acetylcholine-induced vasodilation, demonstrating the dependence of endothelial responsiveness on membrane lipid integrity. The observed hypoxia-induced loss of endothelial cholesterol facilitates TRPV4–BK signaling as an adaptive mechanism to maintain vascular dilation under conditions of reduced oxygen availability [287]. Studies in rat femoral arteries further demonstrate that hypoxia reduces endothelial cholesterol, thereby augmenting TRPV4-mediated vasodilation, whereas cholesterol replenishment dampens this response [288]. Such evidence underscores the importance of cholesterol in maintaining vascular homeostasis. Beyond the vasculature, cholesterol homeostasis is also tightly linked to trabecular meshwork mechanotransduction. The interplay between membrane tension, cholesterol content, TRPV4 signaling, and calcium handling points to a highly dynamic system of mechanical sensing. While cholesterol may serve a protective function by buffering TM cells against hypertension-induced mechanical stress, this adaptive capacity is likely impaired in conditions associated with cholesterol dysregulation, including glaucoma, diabetic retinopathy, Niemann–Pick disease, and macular degeneration [222].

Caires, Sierra-Valdez [27] further emphasize the bidirectional interplay between lipid metabolism and TRPV4-mediated mechanotransduction. ω-3 PUFAs have been shown to modulate TRPV4 function through plasma membrane remodeling rather than direct ligand binding. Enrichment of cellular membranes with ω-3 PUFAs increases membrane fluidity and reduces bending stiffness, thereby attenuating TRPV4-mediated Ca^2+^ influx and mechanosensitivity. In endothelial cells, this lipid-induced modulation dampens excessive mechanotransduction and inflammatory signaling. These findings establish TRPV4 as a lipid-sensitive mechanosensor whose activity can be tuned through dietary membrane remodeling, highlighting nutritional lipid supplementation as a potential therapeutic strategy in vascular and inflammatory disorders.

### 5.5. Lipid Regulation of Mechanotransduction in Cancer

Within the expanding landscape of lipid–channel interactions in cancer, cholesterol has emerged as a key regulator of TRPM7-dependent signaling in prostate tumor progression. Although cholesterol is known to promote cell proliferation and migration, its mechanistic link to ion channel activity has only recently been clarified. Evidence demonstrates that cholesterol enhances Ca^2+^ influx through TRPM7, leading to activation of AKT and ERK signaling pathways that drive prostate cell proliferation [289].Cholesterol-induced Ca^2+^ entry stimulates calpain activity, resulting in reduced E-cadherin expression and enhanced migratory capacity of prostate cancer cells. Overexpression of TRPM7 further amplifies cholesterol-dependent Ca^2+^ influx, proliferation, and tumor growth, whereas TRPM7 silencing or pharmacological inhibition of cholesterol synthesis with statins significantly attenuates these effects. Clinically, increased TRPM7 expression has been observed in prostate cancer tissues, while statin use correlates inversely with prostate cancer incidence [289]. The cholesterol–TRPM7 axis therefore represents a mechanistic bridge between lipid metabolic reprogramming and prostate cancer progression, highlighting TRPM7 as a potential therapeutic target in cholesterol-associated malignancy.

TRPV4 has been implicated as an active regulator of lipid metabolism in oncogenic settings [27]. In ovarian cancer, enhanced TRPV4-mediated Ca^2+^ influx activates mTORC1 signaling and downstream SREBP1-driven fatty acid synthesis, promoting tumor growth and metabolic reprogramming. Increased lipogenesis reinforces membrane remodeling and supports cancer cell proliferation and survival. This TRPV4–Ca^2+^–mTORC1/SREBP1 signaling axis establishes a feed-forward mechanometabolic loop that drives tumor progression. These findings identify TRPV4 as a critical contributor to cancer-associated lipid remodeling and suggest that targeting TRPV4-mediated mechanotransduction may represent a novel therapeutic strategy to disrupt oncogenic metabolic adaptation [290].

### 5.6. Lipid Regulation of Mechanotransduction in Liver Disorders

Within the broader framework of lipid–channel interactions, TRPC5 has emerged as a metabolically sensitive ion channel linking lipid dysregulation to hepatic pathology. Given that TRPC5 is modulated by endogenous lipids implicated in cholestasis [291], it has been proposed that this channel contributes directly to the pathogenesis of cholestatic liver disease [292]. In models of cholic acid (CA)-induced cholestasis, hepatic phospholipid metabolism is profoundly disrupted, leading to dyslipidaemia in a TRPC5-dependent manner. Notably, TRPC5 knockout mice exhibit significantly attenuated liver pathology under cholestatic conditions, supporting a functional role for TRPC5 in mediating local liver injury. These findings indicate that lipid-mediated activation of TRPC5 constitutes a mechanistic link between metabolic imbalance and cholestasis-induced hepatic damage [292]. The evidence positions TRPC5 within the lipid–channel axis that integrates membrane lipid remodeling with ion channel signaling in liver disease. Targeting TRPC5 or modulating its lipid sensitivity may therefore represent a promising therapeutic strategy for cholestasis and related metabolic liver disorders.

### 5.7. Lipid Regulation of Mechanotransduction in Neurological Disorders

TREK-1 functions as a lipid-sensitive potassium channel that connects membrane lipid signaling to neuroprotection in ischemic brain injury. As a member of the K2P channel family, it is highly expressed in the central nervous system and is activated by mechanical stimuli, temperature, pH, voltage, and polyunsaturated fatty acids, including arachidonic acid [293]. Polyunsaturated fatty acids and lysophospholipids are known to protect the brain against global ischemia [294] and because both lipid classes activate TREK-1, the channel has been proposed as a direct mediator of lipid-induced neuroprotection. Buckler and Honoré [295] demonstrated that preserving arachidonic acid availability, TREK-1 remains resistant to hypoxia and is robustly activated by arachidonic acid even at very low oxygen tensions. Moreover, hypoxia does not significantly alter basal, 2,4,6-trinitrophenol-, or acid-stimulated TREK-1 currents. The evidence positions TREK-1 within the lipid–channel axis that integrates membrane lipid remodeling with ion channel signaling in neurological disease. Targeting TREK-1 or modulating its lipid sensitivity may therefore represent a promising therapeutic strategy for stroke and other hypoxic–ischemic brain disorders [295].

Within this lipid–channel framework, TRPC5 function is critically dependent on sphingolipid-mediated membrane organization [296]. As key structural components of lipid rafts, sphingolipids regulate membrane fluidity, protein–lipid interactions, and channel trafficking [296]. In neurons, TRPC5 localizes to cholesterol- and sphingolipid-rich microdomains, where raft integrity is essential for proper membrane targeting and channel activity. Inhibition of sphingolipid synthesis or disruption of lipid rafts markedly impairs TRPC5 localization and reduces Ca^2+^ influx, while lipidomic analyses confirm that balanced sphingomyelin and glycosphingolipid levels are required to maintain channel function. These findings identify TRPC5 as a sphingolipid-sensitive channel governed by membrane microdomain integrity. Disruption of sphingolipid metabolism may therefore destabilize TRPC5 anchoring and broader TRP channel regulation, providing a mechanistic link between lipid metabolic imbalance and diverse neurological phenotypes. Targeting membrane lipid composition could thus represent a promising therapeutic strategy in neuropsychiatric and neurodegenerative disorders [296].

## 6. Conclusions

In this review, we have synthesized current understanding of MSCs and highlighted the diverse ways in which lipid species influence their structure, gating behavior, and downstream signaling. A consistent theme emerging across studies is that lipid–MSC interactions operate through both direct molecular contacts and indirect modulation of membrane mechanics. Specific lipids, such as cholesterol, phosphoinositide, and polyunsaturated fatty acids, can interact with defined channel regions to alter conformational dynamics, ion selectivity, or open probability. In parallel, lipid-driven changes in membrane tension, curvature, stiffness, and microdomain organization profoundly shape the mechanical forces transmitted to MSCs. Together, these mechanisms enable cells to calibrate mechanotransduction with remarkable sensitivity to their mechanical, metabolic, and signaling environment.

The structural complexity and rapid turnover of cellular lipids indicate that the repertoire of lipid modulators of MSCs is far from complete. Accumulating evidence also highlights strong context dependence, whereby the same lipid species can exert distinct or even opposing effects on a given channel depending on cell type, metabolic state, or local membrane composition. This dynamic lipid–MSC interplay is increasingly implicated in a wide range of physiological and pathological processes, including vascular tone regulation, immune cell activation, neurodegeneration, tumor progression, and musculoskeletal dysfunction. Accordingly, a deeper mechanistic understanding of lipid–MSC relationships holds substantial therapeutic promise. Potential strategies include targeting lipid metabolic pathways, developing lipid-inspired channel modulators, and exploiting diet-induced shifts in membrane composition to fine-tune mechanosensitive signaling.

Despite significant recent advances, several key questions remain unresolved, and addressing them will be essential for translating lipid–MSC biology into predictive frameworks and therapeutic interventions.

- Define lipid-binding sites and gating mechanisms with atomic precision. Advances in cryo-electron microscopy, molecular dynamics simulations, and chemical lipid probes will be critical for mapping transient lipid–protein interactions and determining how specific lipid species stabilize distinct channel conformations.
- Characterize lipid heterogeneity in living membranes with high spatial and temporal resolution. Super-resolution imaging approaches, combined with mass spectrometry–based lipidomics, are needed to reveal how mechanical stress, metabolic cues, and signaling events dynamically remodel membrane nanodomains in real time.
- Systematically investigate the context dependence of lipid effects. Comparative studies across cell types, developmental stages, and metabolic states will help explain why identical lipids can modulate the same channel in divergent ways under different physiological conditions.
- Explore therapeutic strategies targeting lipid–MSC coupling. Promising avenues include lipid-based small molecules, selective manipulation of local membrane composition, and metabolic interventions designed to tune MSC sensitivity with high specificity.

Progress in these areas will deepen our understanding of the fundamental principles governing mechanotransduction and may ultimately enable the development of innovative, mechanism-based therapies that exploit the interplay between membrane biophysics, lipid metabolism, and mechanosensitive ion channel function.

## Figures and Tables

**Table 1:** Major families of mechanosensitive ion channels and their defining characteristics. The table summarizes representative members, gating mechanisms, ion selectivity, and principal physiological functions of major mechanosensitive ion channel families. These channels are widely distributed across organisms from bacteria to mammals and mediate diverse processes including osmotic protection, touch sensation, hearing, vascular tone regulation, inflammation, thermosensation, and acid sensing.

Family	Representative Members	Gating Mechanism	Ion Selectivity	Major Physiological Roles
Piezo family	Piezo1, Piezo2	Primarily force-from-lipid; modulated by cytoskeletal and ECM coupling (force-from-filament contribution)	Non-selective cation (Ca^2+^, Na^+^, K^+^, Mg^2+^)	Large, trimeric, non-selective cation channels directly gated by membrane tension. Critical for touch sensation, blood flow sensing.
MscL/MscS	MscL, MscS	Direct bilayer tension sensing	Non-selective	Bacterial osmotic protection
K2P	TREK-1, TREK-2, TRAAK	Membrane stretch and lipid-dependent gating	K^+^ selective	Resting membrane potential, thermosensation
TRP	TRPV4, TRPP2, TRPC1, TRPA1	Polymodal (mechanical + chemical + lipid regulation)	Non-selective cation	Vascular tone, inflammation, mechanosensation
OSCA/TMEM63	OSCA1.1, TMEM63A	Direct tension-activated	Non-selective cation	Osmosensing (plants), mechanosensation (mammals)
MET (Hair Cell)	TMC1, TMC2 (complex components)	Tip-link mediated mechanical gating	K^+^/Ca^2+^ permeable	Auditory mechanotransduction
DEG/ENaC	MEC-4, ASICs	Mechanically modulated/acid activated	Na^+^ selective	Touch (invertebrates), acid sensing (vertebrates)

**Table 2:** Regulation of lipids on mechanosensitive ion channels. The table synthesizes peer-reviewed evidence on lipid regulation of mechanosensitive and related ion channels, listing the lipid species tested, their molecular targets (specific ion channels), observed effects on channel function, proposed mechanisms, and experimental models used. Methods indicate the primary approaches for functional or structural assessment (e.g., patch-clamp configurations, Ca^2+^ imaging, or advanced microscopy).

Lipids	Target in Channel	Effect on Channel Function	Mechanism	Models	Methods	Reference
Cholesterol	Piezo1, Piezo2	Cholesterol removal changed membrane mechanics and reduced mechanosensitivity in a stomatin (STOML3) dependent manner	STOML3 interacts with cholesterol-rich domains to facilitate force transfer	DRG neurons from male C57/Bl6N and STOML3−/− mice; N2A mouse cells	Whole-cell patch clamp	[104]
	Piezo1	Removal of cholesterol enhanced the diffusion of Piezo1-GFP clusters and altered their distribution	Redistribution of Piezo1 nanodomains within the plasma membrane	HEK293T cells transfected with Piezo1-GFP	STORM TIRF	[93]
	Piezo1	Cholesterol depletion delayed activation and slowed inactivation		HEK293T cells transfected with Piezo1-GFP; N2A cells	Cell-attached patch-clamp	[93]
	Piezo1	Loss of cholesterol reduced Yoda1 activation; cholesterol enrichment lowered current amplitude	Possible direct lipid–channel interaction or raft disruption altering Piezo1 distribution	HEK293T transfected with human Piezo1; HUVEC	Intracellular Ca^2+^ measurement by Flexstation, outside-out patch-clamp	[102]
	Piezo1	Cholesterol loading inhibited Piezo1 currents, while depletion enhanced activity		HEK Piezo1del transfected with Piezo1	Cell-attached patch-clamp	[107]
	Piezo1	Cholesterol application increased Ca^2+^ transients, abolished by Piezo1 inhibition/knockdown		Melanoma cells	Ca^2+^ indicator (GCaMP) fluorescence	[108]
Oxidized cholesterol 7-KC	Piezo1	Chronic 7-KC enhanced Yoda1 response, increased mechanosensitive currents, slowed inactivation/deactivation	Lower membrane order facilitates Piezo1 gating	Mouse bone marrow-derived macrophages (BMDMs); Mouse macrophage cell line RAW; HEK Piezo1del transfected with Piezo1	Cell-attached patch-clamp; Fura-2 calcium imaging	[107]
Phospholipid PS	Piezo1	Phospholipid flippase-mediated translocation of cell surface-exposed PS is required for Piezo1 activation	Lipid asymmetry maintains Piezo1 activity	C2C12 cells	Fura-2 calcium imaging	[114]
Phospholipid PIP2	Piezo1	Depletion suppressed Piezo1 currents		HEK293 cells transfected with Piezo1	Inside-out patch-clamp	[124]
	Piezo1	Mutation at the Δ4K site (a potential PIP2–Piezo1 binding region) slowed Piezo1 inactivation	Direct binding of PIP2 to Piezo1	HEK Piezo1del transfected with Piezo1	CG MD simulation; cell-attached patch-clamp	[101]
	Piezo1	Multiple binding sites redundantly stabilize Piezo1 function	PIP2 engages basic residues across intracellular surfaces	HEK 293 cells transfected with the GFP-tagged mouse Piezo1	All-atom (AA) MD simulation; Whole-cell patch clamp	[130]
	Piezo1	PIP2 depletion enhanced Piezo1 sensitivity	PIP2 stabilizes interblade handshake; handshake dampens mechanosensitivity	HEK 293 cells transfected with hPiezo1 or mutant hPiezo1	CG MD simulation; outside-out patch-clamp; intracellular Ca^2+^ measurement by Flexstation	[131]
Phospholipid PA	Piezo2	Intracellular delivery of PA inhibited Piezo2 activity	TMEM120A-mediated lipid remodeling reduced Piezo2 currents	N2A-Piezo1-KO cells transiently transfected with Piezo2-GFP or Piezo1-GFP	Whole-cell patch clamp	[133]
Phospholipid LPA	Piezo2	Intracellular delivery of LPA inhibited Piezo2 activity	TMEM120A-dependent lipid regulation	N2A-Piezo1-KO cells transiently transfected with Piezo2-GFP or Piezo1-GFP	Whole-cell patch clamp	[133]
Phospholipid derivative ccPA	Piezo2	Long incubation of ccPA treatment inhibits both Piezo2-mediated mechanically activated current	TMEM120A-dependent lipid regulation	N2A-Piezo1-KO cells transiently transfected with Piezo2-GFP or Piezo1-GFP	Whole-cell patch clamp	[133]
Sphingolipid Sphingomyelin	Piezo1	Altered pressure sensitivity and reduced inactivation	Changes in lipid environment modulate Piezo1 mechanics	Freshly isolated endothelium from second-order branches of mouse mesenteric arteries, HEK T-rex transfected with Piezo1	Outside-out patch clamp; intracellular Ca^2+^ measurement by Flexstation	[136]
Sphingolipid Ceramide	Piezo1	Abolished inactivation	SMPD3-dependent lipid remodeling	Freshly isolated endothelium from second-order branches of mouse mesenteric arteries, HEK T-rex transfected with Piezo1	Outside-out patch clamp; intracellular Ca^2+^ measurement by Flexstation	[136]
Fatty acid MA	Piezo1	Inhibited Piezo1 activation	MA increased membrane bending stiffness	N2A mouse cells; human microvascular endothelial cells (HMVEC)	Whole-cell patch clamp	[18]
	Piezo2	MA suppressed Piezo2 current	Increased membrane rigidity	N2APiezo1−/− cells transfected with Piezo2; human Merkel cell carcinoma cell line (MCC13), mouse dorsal root ganglia (DRG) neurons	Whole-cell patch clamp	[26]
Fatty acid AA	Piezo1	Shortened inactivation time	Reduced bilayer stiffness	N2A mouse cells; HMVEC	Whole-cell patch clamp	[18]
	Piezo1	Decreased channel sensitivity		Chondrocytes	Ca^2+^ imaging using confocal microscopy	[141]
Fatty acid EPA	Piezo1	Faster inactivation	Reduced membrane bending rigidity	N2A mouse cells; HMVEC	Whole-cell patch clamp	[18]
	Piezo1	Reduced Ca^2+^ response to compression or Yoda1		Chondrocytes	Ca^2+^ imaging using confocal microscopy	[141]
Fatty acid DHA	Piezo1	Prolonged inactivation	Lowered bilayer stiffness	N2A mouse cells; HMVEC	Whole-cell patch clamp	[18]
	Piezo1	DHA supplementation increased Piezo1-GFP sensitivity to pressure, prolonged the latency of response and abolished activation	Incorporated into phospholipids, altering bilayer mechanics	HEK293T cells transfected with Piezo1-GFP	Cell-attached patch-clamp	[93]
	Piezo1	Reduced Ca^2+^ response to compression or Yoda1		Chondrocytes	Ca^2+^ imaging using confocal microscopy	[141]
	Piezo1	Acute treatment reversibly inhibited Piezo1 currents; chronic treatment slowed inactivation		BMDMs; RAW; HEK Piezo1del transfected with Piezo1	Inside-out patch-clamp; outside-out patch-clamp	[107]
Fatty acid LA	Piezo1	Reduced Ca^2+^ response to compression or Yoda1		Chondrocytes	Ca^2+^ imaging using confocal microscopy	[141]
Fatty acids Oleic acid	Piezo 1	Reduced current amplitude under pressure		HEK Piezo1del transfected with Piezo1	Inside-out patch-clamp	[107]
Phospholipid PA	TREK-1	PA strongly activated TREK-1		COS cells	Inside-out patch-clamp	
	TREK-1	PA activated TREK-1	a PA lipid inserts its hydrocarbon tail into a pocket behind the selectivity filter, causing a structural rearrangement that recapitulates mutations and pharmacology known to activate TREK1		Cryo-EM	[186]
Phospholipid PIP2	TREK-1	Cytosolic PIP2 stimulated TREK-1 channel activity	a cluster of basic residues in the proximal C-terminus adjacent to TM4 as a potential PIP2-binding site	COS cells	Inside-out patch-clamp	[180]
	TREK-1	Increasing PIP2 after activation inhibits TREK-1		transfected COS cell expressing TREK-1.	Inside-out patch-clamp	[181]
	TREK-1	PIP2 inhibit TREK-1	PIP2 directly binds to TREK-1 and competes with lipid agonists PA and phosphatidylglycerol (PG) in purified liposomes		Ion flux assays	[183]
Cholesterol	TRPC1	Colocalization of TRPC1 with caveolin-1 was reduced by depletion of cholesterol	Cholesterol influences vascular reactivity to ET-1 by affecting the caveolar localization of TRPC1	caudal artery sections	Immunofluorescence	[215]
	TRPC3	Cholesterol activates a cation conductance. Membrane loading with cholesterol promotes basal Ca^2+^ entry into in TRPC3-expressing cells		HEK-293 cells stably transfected to express TRPC3 channels	Whole-cell patch clamp; intracellular Ca^2+^ measurement	[217]
	TRPC6	Podocin binds cholesterol, and this binding regulates TRPC6 activity		Xenopus oocytes	Patch clamp	[220]
	TRPV4	Cholesterol depletion increased the amplitude of TRPV4 agonist-induced Ca^2+^ signals	Lowering the levels of free membrane cholesterol facilitated TRPV4 activation and promoted cytoskeletal polymerization	Trabecular meshwork (TM) cells were dissected from three eyes of donors	Ca^2+^ imaging	[222]
	TRPV1	Augmentation of cholesterol reduced TRPV1 currents	Cholesterol inhibits TRPV1 by binding to specific sites along the S5 helix, having a putative CRAC motif	HEK293 cells transfected with TRPV1	Inside-out patch-clamp	[226]
	TRPV4	TRPV4 is expressed in Mesenchymal stem cells, and the localization of TRPV4 in lipid rafts is dependent on temperature and cholesterol	Cholesterol directly binds and stabilizes TRPV4 (TM4–Loop4–TM5)	Mesenchymal stem cells	Confocal microscopy	[223]
DAG	TRPC3 TRPC6	DAG activated TRPC3 and TRPC6 in a membrane-delimited way.		CHO-K1 cells	Inside-out patch-clamp; intracellular Ca^2+^ measurement	[218]
PIP2	TRPV2	Reduction of PIP2 contributes to Ca^2+^-dependent desensitization of TRPV2 channels		F-11 cells were transfected with TRPV2	Confocal microscopy; Whole-cell patch clamp	[230]
	TRPV4	Depletion of PIP2 levels prevented TRPV4 channel activation by physiological stimuli		HeLa cells transfected with TRPV4; ciliated epithelial cells	Inside out patch-clamp; ratiometric Ca^2+^ recordings	[231]
	TRPV4	PIP2 suppressed TRPV4 activity	PIP2 binds to the N-terminus of TRPV4 exerts an inhibitory effect on the channel activity	HEK-293 cells transfected with TRPV4	Whole-cell patch clamp	[232]
	TRPV4	PIP2 depletion promoted TRPV4 channel activity		Capillary endothelial cells	Whole-cell patch clamp	[233]
Fatty acids PUFA	TRPV4	PUFAs are required for TRPV4 function		C.elegans	Behavioural assays	[283]
Fatty acids Oleate	TRPV4	Increasing intracellular lipid content increases TRPV4 activity		Lung microvascular endothelial cells (MVECs)	Ca^2+^ imaging	[236]
Fatty acids AA	ASICs	AA potentiated ASIC currents	AA binds with the ASIC directly	dorsal root ganglion neuron (DRG)	Whole-cell patch-clamp	[277]
	ASIC3	AA potentiated the pH 7.2-evoked ASIC3 current	The potent effect of AA on the ASIC3 current essentially results from a shift in the pH dependence of activation towards less acidic values	Skin DRG	Whole-cell patch clamp	[278]
Phospholipids LPC	ASIC3	LPC activated ASICs		HEK293 and F-11 cells express human or rat ASIC3	Whole-cell patch clamp	[279]
Fatty acids AA	ASIC3	AA activated ASICs		HEK293 and F-11 cells express human or rat ASIC3	Whole-cell patch clamp	[279]
Fatty acids PUFA and their derivatives	ASIC3	Potentiated ASIC3 currents	increase ASIC3 currents by shifting the pH dependence of activation and stabilizing the open state of the channel	Chinese hamster ovary (CHO) cells expressing ASIC3	Whole-cell patch clamp	[280]
Cholesterol	ENaC	Depletion of cholesterol reduced ENaC activity	decreasing PIP2 in the microvilli. PIP2 can directly stimulate ENaC	A6 cells	Single-channel patch-clamp	[281]

## Data Availability

No new data were created or analyzed in this study. Data sharing is not applicable to this article.

## Abbreviations

The following abbreviations are used in this manuscript:

7-KC	7-Ketocholesterol
AA	All-atom
AA	Arachidonic acid
AD	Alzheimer’s disease
ADPKD	Autosomal dominant polycystic kidney disease
AFM	Atomic force microscopy
ARD	Ankyrin repeat domain
ASICs	Acid-sensing ion channels
BOHB	β-hydroxybutyrate
CaPLSase	Ca^2+^-activated phospholipid scramblase
CARC	Cholesterol-recognition amino acid consensus
Cav-1	Caveolin-1
ccPA	carbocyclic PA
CG	Coarse-grained
CH	Chronic hypoxia
CL	Cardiolipin
CNS	Central nervous system
cryo-EM	Cryo-electron microscopy
DAG	Diacylglycerol
DEE	Developmental and epileptic encephalopathy
DEG	degenerin
DHA	Docosahexaenoic acid
DOPC	1,2-dioleoyl-sn-glycero-3-phosphocholine
DOPE	1,2-dioleoyl-sn-glycero-3-phosphoethanolamine
DRG	Dorsal root ganglion
ECM	Extracellular matrix
EDHF	Endothelium-derived hyperpolarizing factors
ENaC	Epithelial sodium channel
eNOS	Endothelial nitric oxide synthase
EPA	Eicosapentaenoic acid
ER	Endoplasmic reticulum
FFF	Force-from-filament
FFL	Force-from-lipid
FID	Flow-induced dilation
FSGS	Focal segmental glomerulosclerosis
GFP	Green fluorescent protein
HEK	Human embryonic kidney
HMVEC	Human microvascular endothelial cell
hPiezo1	Human Piezo1
HUVECs	Human umbilical vein endothelial cells
HX	Hereditary xerocytosis
ICD	Intracellular domain
IL	Intracellular linker
INF2	Inverted formin-2
iPSC	Induced pluripotent stem cell
K2P	Two-pore domain potassium
KO	Knockout
LA	Linoleic acid
LDT	Locally distributed tension
LH	Long helix
LPA	Lysophosphatidic acid
LPC	Lysophosphatidylcholine
LPS	Lipopolysaccharides
LysoPA	Lyso-phosphatidic acid
LysoPC	Lyso-phosphatidylcholine
LysoPS	Lyso-phosphatidylserine
MA	Margaric acid
MCC	Merkel cell carcinoma
MD	Molecular dynamics
MERCs	Mitochondria–ER contact sites
MET	Mechanoelectrical transduction
mPiezo1	Mouse Piezo1
MscL	mechanosensitive channel large conductance
MSCs	Mechanosensitive ion channels
MscS	Mechanosensitive channel small conductance
Mt	Mycobacterium tuberculosis
MVECs	Microvascular endothelial cells
MβCD	Methyl-β-cyclodextrin
N2A	Neuro 2A
NO	Nitric oxide
NompC	No mechanoreceptor potential C
NSCs	Neural stem cells
NSmase	Neutral sphingomyelinase
OSCA	Osmolality-induced [Ca^2+^] increase
PA	Phosphatidic acid
PAH	Pulmonary arterial hypertension
PC2	Polycystin-2
PE	Phosphatidylethanolamine
PG	Phosphatidylglycerol
PI	Phosphatidylinositol
PI3K	Phosphoinositide 3-kinase
PIP2	Phosphatidylinositol 4,5-bisphosphate
PKC	Protein kinase C
PLC	Phospholipase C
PLD	Phospholipase D
POPC	1-Palmitoyl-2-oleoyl-sn-glycero-3-phosphocholine
PS	Phosphatidylserine
PUFAs	Polyunsaturated fatty acids
RBCs	Red blood cells
SCA	Sickle cell anemia
SCD	Sickle Cell Disease
SH	Short helix
SMPD3	Sphingomyelinase 3
STOML3	Stomatin-like protein 3
TM	Transmembrane
TMEM	Transmembrane protein
TMHS	Tetraspan membrane protein of hair cell stereocilia
TMIE	Transmembrane inner ear protein
TRP	Transient receptor potential
VSMCs	Vascular smooth muscle cells
